# Sugarcane Giant Borer Transcriptome Analysis and Identification of Genes Related to Digestion

**DOI:** 10.1371/journal.pone.0118231

**Published:** 2015-02-23

**Authors:** Fernando Campos de Assis Fonseca, Alexandre Augusto Pereira Firmino, Leonardo Lima Pepino de Macedo, Roberta Ramos Coelho, José Dijair Antonino de Sousa Júnior, Orzenil Bonfim Silva-Junior, Roberto Coiti Togawa, Georgios Joannis Pappas, Luiz Avelar Brandão de Góis, Maria Cristina Mattar da Silva, Maria Fátima Grossi-de-Sá

**Affiliations:** 1 Embrapa Recursos Genéticos e Biotecnologia, Brasília, Distrito Federal, Brazil; 2 Universidade de Brasília, Brasília, Distrito Federal, Brazil; 3 Universidade Federal do Rio Grande do Sul, Porto Alegre, Rio Grande do Sul, Brazil; 4 Universidade Católica de Brasília, Brasília, Distrito Federal, Brazil; 5 Usina triunfo, Maceió, Alagoas, Brazil; Institute of Plant Physiology and Ecology, CHINA

## Abstract

Sugarcane is a widely cultivated plant that serves primarily as a source of sugar and ethanol. Its annual yield can be significantly reduced by the action of several insect pests including the sugarcane giant borer (*Telchin licus licus*), a lepidopteran that presents a long life cycle and which efforts to control it using pesticides have been inefficient. Although its economical relevance, only a few DNA sequences are available for this species in the GenBank. Pyrosequencing technology was used to investigate the transcriptome of several developmental stages of the insect. To maximize transcript diversity, a pool of total RNA was extracted from whole body insects and used to construct a normalized cDNA database. Sequencing produced over 650,000 reads, which were *de novo* assembled to generate a reference library of 23,824 contigs. After quality score and annotation, 43% of the contigs had at least one BLAST hit against the NCBI non-redundant database, and 40% showed similarities with the lepidopteran *Bombyx mori*. In a further analysis, we conducted a comparison with *Manduca sexta* midgut sequences to identify transcripts of genes involved in digestion. Of these transcripts, many presented an expansion or depletion in gene number, compared to *B. mori* genome. From the sugarcane giant borer (SGB) transcriptome, a number of aminopeptidase N (APN) cDNAs were characterized based on homology to those reported as Cry toxin receptors. This is the first report that provides a large-scale EST database for the species. Transcriptome analysis will certainly be useful to identify novel developmental genes, to better understand the insect’s biology and to guide the development of new strategies for insect-pest control.

## Introduction

Lepidopteran stem borers are economically important agricultural insect pests that cause severe damage to sugarcane crops worldwide. Because of its endophytic behavior, the use of chemical pesticides is mostly ineffective and requires manual labor, which is time consuming and increases the cost [1,2]. Historically, synthetic insecticides, which are usually aerially applied, were used against moth borers accomplishing moderated success. Furthermore, repetitious applications may contribute to environmental pollution and generate health problems [3,4]. Alternatives to chemical control in sugarcane crops include transgenes based on the endotoxins produced by the bacterium *Bacillus thuringiensis* (Bt), which is widely used for protection against lepidopterans in cereal crops such as rice and maize [5,6]. There are several lepidopteran insect pests that affect sugarcane worldwide that could be targeted by the expression of Bt toxins in transgenic plants [7]. However, to combat the numerous species of borers involved in the infestation of sugarcane crops, basic and applied research must be conducted to increase our knowledge of the biology and ecology, as well as management of the species.

The sugarcane giant borer *Telchin licus licus* (SGB) (Drury, 1773) (Lepidoptera: Castiniidae) has become a major insect pest in the sugarcane fields of Central and South America [8]. To control infestation, several methods were evaluated, including biological control, mechanical collection and identification of resistant plants, but none of these strategies turned out to be successful. Moreover, the insect spends most of its life cycle, -six to ten months-, feeding inside the plant, and is not easily affected by chemical pesticides [9]. Despite its economic importance, studies with this insect aiming pest control are still at an early stage. At present, no reports demonstrating the establishment of an insect rearing system for this insect is available, thereby hindering the study of its development. To date, a few published reports have discussed about the chemical composition of pheromones [10], body morphology [11], entomopathogenic activity of fungi [12] and *in vitro* evolution of Cry toxins [13]. Recently, 109 mitochondrial DNA sequences were used to determine the origin of invasive subspecies of *T*. *licus* in sugarcane producing regions in Brazil [14] and are now publicly available at the GenBank. The lack of information regarding the insect´s physiology and molecular biology obliged us to make use of data from model insects like *Bombyx mori*, whose genome has been fully sequenced [15] and Expressed Sequence Tags (ESTs) libraries from organisms of the same order [16], hampering cloning and characterization of genes and their expression analysis.

Large scale sequencing through next generation sequencing technologies has effectively increased the number and depth of genomic and transcriptomic data [17–19]. This approach is being applied in entomology for gene discovery [20], gene expression analysis of specific tissues and at different physiological conditions [21], to analyze single nucleotide polymorphisms (SNPs) [22], development studies of resistance to insecticides [23,24], understanding endosymbiosis interactions [25], and to determine specific target genes to be silenced through RNA interference [26]. With the increase of DNA sequence information, new opportunities for analysis are emerging. To date poplar leaf beetle’s (*Chrysomela tremulae*) and tobacco hornworm’s (*Manduca sexta*) midgut transcriptomes have been sequenced [27,28], making it possible to compare data from different organisms and identify new gene families. Likewise, transcriptome analysis can provide an assessment of gene families that can be used in the biotechnological industry as those involved in biomass conversion [29]. Recent reports have demonstrated the presence of genes related to cellulose degradation in some insect species [27], with the possibility of finding similar genes in different organisms.

The insect midgut is involved in several processes, including digestion, immunity and mechanical protection. For these reasons it became an important organ to study [30]. Searching for new genes may help to understand (i) the role of receptors that participate in toxin resistance [31], (ii) ecological traits [32], (iii) disease transmission [33] and (iv) identify which phenotypic alterations may be caused by silencing specific genes [34].

Depending on their feeding habits, insects can regulate gene expression to increase their adaptability to different diets [35]. In contrast to other lepidopteran caterpillars that feed primarily on leaves, the SGB presents an endophytic behavior, feeding within the steam of sugarcane plants, whose composition is richer in sucrose than starch. This characteristic can influence the differential expression of digestive enzymes, leading to a specific intestinal homogenate composition that guarantees the insect´s survival and development within the plant.

The insect digestive system is primarily composed of proteases, a major group of hydrolytic enzymes that participates on digestion [36] and proenzyme activation [37]. These enzymes are also related to developmental processes such as molting and metamorphosis, can act as a regulator of innate immune response [38–41] and are associated with Cry toxin activation and solubilization [42,43]. Several reports have proposed the digestive system as a target for pest population management [44–47], based on the fact that any alterations in the enzyme activity of the intestinal homogenate or the use of protease inhibitors (PIs) can modify the insects’ metabolism and lead to a reduction of nutrient absorption, thus hindering its development [48,49]. Recently, a Kunitz-type protease inhibitor was characterized against insects’ intestinal homogenate, including SGB larvae and a substantial decrease in midgut serine protease activity was observed, although they provided no information concerning the mortality of the insects [50]. In a variety of studies, protease inhibitors have been successfully evaluated, demonstrating that transgenic adoption strategy is an efficient method for insect pest control [51–53].

Among several classes of proteases that are targeted by insect management programs, aminopeptidase N (APN) stands out for its dual characteristic. Besides its importance in digestion of proteins and release of amino acids for cell metabolism, APNs have been studied, together with cadherin-like (BT-R) proteins and alkaline phosphatases (ALP) as putative Cry toxin receptors [54–57]. Silencing APN genes through RNAi demonstrated that the susceptibility of the insect to Cry toxins could be altered [58,59] and that, at least for a few species, the development of resistance to Bt-based pesticides involves the alteration of APN gene expression [60].

The present work describes the construction of an EST dataset of *T*. *licus licus*, a non-model organism, which attacks severely sugarcane crops and for which no information about its molecular biology and physiology is available. To identify a broad range of genes, a normalized whole-body library containing different life stages, including eggs, early-stage larvae, late-stage larvae, prepupae, pupae and adults (both male and female) was sequenced using the 454 sequencing system. This research focused on describing the transcripts involved in the expression of digestive enzymes and highlight potential candidates to be used for pest control. Over 24,000 contigs were obtained from which a selection is currently investigated to get insight into the insect’s biology and to develop new strategies for a more effective pest management.

## Materials and Methods

### Insects and RNA extraction

The Chico Mendes Institute for Biodiversity Conservation (ICMBIO), through the Biodiversity Information System (SISBIO), authorizes the collection of specimens of Brazilian fauna for research purposes in public and private properties. We obtained a Permanent License for Fauna Collection, n° 34833-1, issued in 07/05/2012. This License authorized the Brazilian Agricultural Research Corporation (Embrapa) and its researchers to collect the specimens used in this study. No endangered or protected species were involved in this research.

Late-stage larvae, prepupae, pupae and adults were collected directly from infested sugarcane fields of Triunfo sugarcane mill, located in Maceió, Alagoas state (AL). Females were kept in entomological cages for oviposition. The eggs were collected and maintained at 28 ± 2°C, 70 ± 10% relative humidity and a photoperiod of 12:12 h (Light: Dark) until hatching. The larvae were individualized and reared with sugarcane pieces. A pool of insects of the same stage was frozen in liquid nitrogen prior to RNA isolation.

Total RNA was extracted separately from each insect stage, eggs, early-stage larvae, late-stage larvae, prepupae, pupae, and both male and female adults using Trizol Reagent (Invitrogen, Life Technologies), according to the manufacturer’s protocol. RNA was treated with RNAse-free DNase I (Ambion, Life Technologies) at 37°C for 30 minutes, according to the manufacturer’s protocol.

### cDNA Library Normalization and Pyrosequencing

A pool of 30 μg of all insect stages total RNA was sent to Eurofins MWG Operon, in Huntsville, AL, USA (http://www.eurofinsdna.com/) to synthesize a cDNA library.

The RNA quality was assessed using an Agilent 2100 Bioanalyzer prior to cDNA library construction. Full-length, enriched cDNAs were generated using the SMART PCR cDNA synthesis kit (BD Clontech) following the manufacturer’s protocol. The resulting double-stranded cDNAs were normalized using the Kamchatka crab duplex-specific nuclease method (Trimmer cDNA normalization kit, Evrogen) to prevent over-representation of the most common transcripts [61]. The normalized transcripts were submitted to a half-plate run using the 454 pyrosequencing, GS FLX Titanium technology, according to the protocols provided by the manufacturer (Roche 454 Life Sciences). Raw data from the sequencing run were submitted to the Sequence Read Archive repository of the National Center for Biotechnology Information (NCBI) under accession number SRR1204999.

### Data Pre-Processing

Pre-processing of pyrosequencing reads for quality and adaptor trimming was first performed using the runAssembly function of Newbler version 2.5.3 using -cdna and -tr options. This latter option was used to output the trimmed reads. *Est2assembly* 1.03 platform was used on previously trimmed reads to prepare data for the assembler [62]. Contaminant sequences (prokaryotic, viral and mitochondrial sequences) were removed after BLAST analysis using locally prepared databases. Repetitive sequence identification and Poly A/T tail trimming was performed using RepeatMasker and standardizing options in the est2assembly preprocessed pipeline.

### Assembly, Annotation and Gene Ontology (GO)

As there were no SGB data or DNA sequences of other phylogenetically related organisms, the contigs were *de novo* assembled using MIRA v3.3.0.1 [63]. The resulting contigs were submitted to the Transcriptome Shotgun Assembly repository of the National Center for Biotechnology Information (NCBI) under accession number SRR1204999. Unique sequences were determined by similarity searches using the BLASTx tool. Functional annotation by GO terms (http://www.geneontology.org), InterPro entries (InterProScan; http://www.ebi.ac.uk/Tools/pfa/iprscan/), enzyme classification codes (EC) and metabolic pathways (KEGG, Kyoto Encyclopedia of Genes and Genomes; http://www.genome.jp/kegg/) were determined using the Blast2GO software suite v2.4.3 (http://www.blast2go.org) [64]. Sequences were submitted to the NCBI protein nr databank via BLASTx, with an e-value threshold of 1e^-5^. False Discovery Rate (FDR) was used at probability level of 0.05%. GO terms were improved with the ANNEX tool [65], followed by GOSlim tool available at Blast2GO (goslim_generic.obo) [66]. Combined graphs were constructed at level 2, for Biological Process, Molecular Function and Cellular Component categories. Enzymatic classification codes and KEGG metabolic pathways were generated by direct mapping of GO terms, with their respective ECs. InterPro searches were performed remotely from Blast2GO on InterProEBI server.

### De novo Contig Assembly Quality Assessment

Contig sequences after assembly were analyzed for biases already known to be induced by the methods used to generate cDNA libraries accordingly to the NGS technologies requirements (i.e. fragmentation and synthesis) that could impact the quality and further functional analysis. To do this we carried a two-tier analysis of the sequencing coverage at base pair-level by: a) analyzing the alignments of the EST sequences on the reference genome of the related *B*. *mori* for all the annotated genomic loci representing protein-coding regions; b) analyzing the alignments of the EST sequences back on the 13,562 contigs that we did not found any functional information. For a) we use the GFF formatted file distributed along the *B*. *mori* genome (SilkDB 2.0) describing the genomic position of the exons for each one of the 16,823 annotated transcripts. In summary we use the GMAP program with the cross-species option to gather the read depth coverage at base pair-level from the alignment of the 61,775 ESTs on 1,009 putative protein-coding genomic loci with RPKM > 0.125. This cut-off value of RPKM was used accordingly as reported previously in a RNA-Seq study aimed to determine the minimum detectable level of expression below which expression can be considered as noise [67]. At this cut-off the mean depth of the coverage of data from the remaining aligned ESTs was 29.8x for the average sampled genes. For b) in the absence of reference gene structure we extracted, for each contig sequence, coverage data from the regions spanning 5% to 15% and 85% to 95% of the putative transcript, by length. These two coverage regions represent in our analysis the ends (3' UTR/ 5' UTR) of the transcript. The first and last 5% of the sequence, by length, was excluded to avoid artifacts from the assembly process. The remaining region, spanning 15% to 85% represent the middle of the cDNA strand. This approach rely largely in the processivity analysis for RNA-Seq coverage data described by Lahens and coworkers (2014) [68]. Both analysis for coverage of data were conducted using BEDTools program [69]. The geneBody_coverage module of the RSeQC program was used to infer if ESTs coverage along the reference models is uniform and if there is any bias towards the ends or the mid-range of the sequences [70].

### Sequence Comparison with *M. sexta* and *B. mori* data

Contig sequences were BLASTed against 13,828 midgut DNA sequences of *M*. *sexta* that were obtained from InsectaCentral [71]. Sequences with e-value score above the cut-off (1e^-3^) were selected. The BLAST results were organized by InterPro terms and description; and the most frequent results were listed. Digestive enzyme sequences were searched by annotation, sequence similarities and InterPro terms.

Digestive enzyme sequences were submitted to the online tool TRAPID [72] for comparative analysis of SGB transcripts and the *B*. *mori* genome. The OrthoMCL-DB proteome database was used as a reference. Protein family groups were identified and organized accordingly to the gene expansion or depletion information.

### 5’ RACE of APN1

Sequencing of aminopeptidase N1 was completed by Rapid Amplification of cDNA Ends (RACE). Neonate larvae of SGB were frozen in liquid nitrogen and total RNA was extracted using TRIZOL reagent (Invitrogen, Life Technologies). An aliquot was applied on 1% agarose gel and subjected to electrophoresis to analyze the integrity of the RNA sample. Quantification was performed using a Nanovue Plus Spectrophotometer (GE life sciences). After incubation with DNase I (Ambion, Life Technologies), 5 μg total RNA was used for cDNA synthesis using M-MLV reverse transcriptase (Invitrogen, Life Technologies) and gene specific primer 1. Addition of a 3’ hydroxyl terminus tail was made using terminal transferase enzyme (New England BioLabs). All experiments were performed accordingly to manufacture’s protocols. Amplification of DNA fragment was carried out in two rounds of polymerase chain reaction (PCR). The first round consisted of 5 μL of cDNA template, 1X PCR buffer, 2 mM MgCl_2_, 0.2 mM dNTP mix, 0.2 μM oligo-dT_30_ adapter primer, 0.2 μM gene specific primer 2, 0.1 units of *Taq* DNA Polymerase (Ludwig Biotec) and deionized water to a final volume of 20 μL. The reaction conditions were: 94°C for 1’, 30 cycles of 94°C for 45”, 60°C for 45” and 72°C for 1’, followed by a final step of 72°C for 5’. The second round of PCR consisted of 1μl (1:20) of the first round template, 1X PCR buffer, 2 mM MgCl_2_, 0.2 mM dNTP mix, 0.2 μM anchor primer, 0.2 μM gene specific primer 3, 0.1 units of *Taq* DNA Polymerase (Ludwig Biotec) and deionized water to a final volume of 20 μL. The reaction conditions were the same as cited previously. Primer sequences are shown in S1 Table. The entire volume was applied on 1% agarose gel and subjected to electrophoresis. DNA fragments were collected from the gel under UV light and purified using the QIAquick Gel Extraction Kit (Qiagen), followed by ligation into PCR 2.1 vector (Invitrogen, Life Technologies). After transformation of OMNIMAX *E*. *coli* cells using Heat Shock method (Invitrogen, Life Technologies) and purification of plasmids by alkaline lysis [73], sequencing was carried out using M13 forward and reverse primers on a ABI377 sequencer (Applied Biosystems, Life Technologies). The DNA sequences were submitted to the dBEST database under accession numbers JZ578314—JZ578318.

### Real-Time PCR Experiments

Transcript levels of serine proteases and APNs were determined in three different tissues (Anterior midgut, Posterior midgut and Carcass) by qPCR using a 7500 Fast Real-Time PCR System (Applied Biosystems, Life Technologies). Insects were dissected under a stereomicroscope (Zeiss Stemi SV6, Jena, Germany) and the tissues directly frozen in liquid nitrogen. A pool of tissues was used for total RNA extraction using Trizol Reagent (Invitrogen, Life Technologies). After DNase I treatment (Ambion, Life Technologies), 1 μg total RNA was used for cDNA synthesis using M-MLV reverse transcriptase (Invitrogen, Life Technologies) and oligo-dT_30_ adapter primer, accordingly to manufacture’s protocol. Reaction mixtures contained 2 μL of cDNA (1:20 diluted), 2.5 μL of Fast SYBR Green Master Mix (Applied Biosystems), 0.2 μM of each primer (S2 Table) and double distilled H_2_O to a final volume of 10 μL. PCR conditions were as follows: 95°C for 20 s, followed by 40 cycles of 95°C for 3 s and 60°C for 30 s. At the end of the program a melting curve for each primer pair (60–94°C read every 0.5°C) was acquired to ensure that only single products were amplified. The SGB glyceraldehyde 3-phosphate dehydrogenase (GAPDH) and 18S ribosomal subunit (RPS18) were used for normalization of qPCR data (S2 Table). Raw data were treated using the online tool qPCR miner (http://www.miner.ewindup.info) [74] to determine primers efficiency and Cq values. The relative expression of each gene was calculated using the qBASE plus program (Biogazelle, Belgium). Three independent quantitative qPCR reactions were carried out per sample and two biological replicates were performed.

### Protease and Aminopeptidase Sequence Analysis

The amino acid sequences of serine proteases and aminopeptidases were obtained by *in silico* translation using TrEMBL (http://www.expasy.ch/tools/dna.html) [75]. Manual screening was carried out to correct mis-assembled contigs and frameshifts, when necessary. Prediction of signal peptides, molecular weight, isoelectric point and glycosylation sites were predicted by using, respectively, SignaIP 4.1, Compute pI/MW, NetNGlyc 1.0 and NetOGlyc 4.0 online tools hosted at ExPaSy: SIB Bioinformatics Resource Portal (http://www.expasy.org/tools/). The GPI anchoring signal was predicted by using PredGPI online tool (http://gpcr2.biocomp.unibo.it/gpipe/index.htm) [76].

### Sequence Alignment and Phylogenetic Analysis

All sequences were aligned using ClustalW2 [77] and edited by BioEdit software v.7 [78]. Phylogenetic analysis were conducted using MEGA v.5 where neighbor-joining trees were constructed with bootstrap of 10,000 replicates and evolutionary divergence calculated by p-distance method [79].

## Results and Discussion

### Sequence Analysis, De Novo Assembly and Annotation

A cDNA library synthesized from a mixture of insect total RNA from different life stages was normalized to reduce over-abundant transcripts. Sequencing was carried out on a half plate of the GS-FLX 454 pyrosequencer, resulting in 653,511 reads with 381,273,406 bp. After bioinformatics preprocessing, 362,412 high-quality reads were obtained and assembled into 23,824 contigs with average depth of coverage of 8.5 sequences per nucleotide position and length from 290 bp to 5,527 bp, with an average of 633 bp (Table 1). A similar pattern of coverage data was observed for other non-model insects that had their transcriptomes sequenced on the 454 Titanium: The non-normalized transcriptome of several life stages of the insect *Anopheles funestus* were pyrosequenced on a half plate of the 454 Titanium generating a 8.3 read per contig coverage [80]. *De novo* transcriptome assembly was performed for the apple maggot (*Rhagoletis pomonella*) and obtained 13.92 reads per contig coverage [32]. The same strategy depicted in this study was recently used by our group to generate an average coverage of 9.58 for the coleopteran *Anthonomus grandis* transcriptome [26]. Sequencing raw data for the present study was deposited in the Short Read Archive of the National Center for Biotechnology Information with accession number SRR1204999.

**Table 1:** Summary of the *Telchin licus licus* transcriptome.

Number of reads before pre-processing	653511
Number of bases before pre-processing	381273406
Average read length before pre-processing	583
Number of reads after pre-processing	362412
Number of bases after pre-processing	140286056
Average read length after pre-processing	387
Number of contigs	23824
Number of bases in contigs	15166298
Average contig length	633
Min. contig length	290
Max. contig length	5527
Average read coverage per contig	8.5
% contigs with at least 1 GO term	18
% contigs with an EC number	0.6
% contigs with at least 1 IPR	16
Contigs with at least 1 BLAST hit against NR	8708
Contigs with no BLAST hits	15116

The assembled sequences (23,824 contigs) were analyzed for similarities with known sequences against non-redundant protein database at NCBI using the BLASTx program of the BLAST suite of tools [81]. At the superior cut-off threshold for blast search set to 1e^-5^, 8,708 contigs (~37%) returned hits against this database. About 60% of the contigs did not show significant sequence similarity at protein-level, reinforcing the findings reported in other studies aiming to explore insect midgut transcriptome [28,82]. The remaining contig sequences (15,116) were inspected for the occurrence of ORFs and domain search querying Pfam-A to provide functional information using Transdecoder program [83]. This procedure resulted in the annotation of 582 additional contigs. Alignment of the contigs to the reference genome of *B*. *mori* [84] using the cross-species option in GMAP program [85] allowed to the recognition of another 605 contigs for which the most probable placement in the genome was coincident with annotated gene model in this related species. Additional search for sequence similarity was carried out using BLASTn program against the NCBI collection of nucleotides (nt), using a stringent e-value cut-off of 1e^-10^ to recognize sequences assembled into contigs in which content was typically associated with untranslated regions resulted in 367 matches. This disjoint conjunct of sequences based on similarities searches resulted in a total of 10,262 contigs (43%) putatively representing reliable set of genes for SGB.

**Fig 1:**
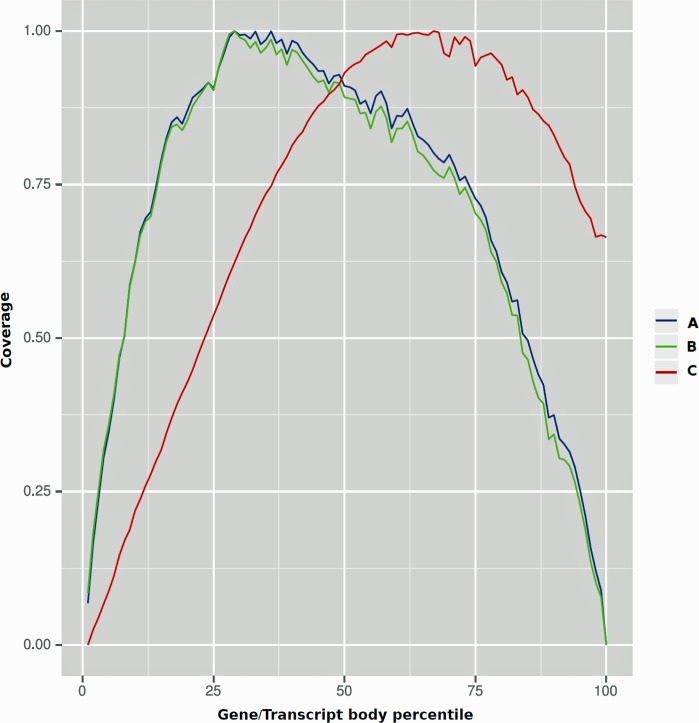
Coverage of data along the gene/transcript length suggests the presence of sequencing bias in the particular set of ESTs. Coverage signals were extracted from BAM files and 100 quantiles were obtained from each transcript in BED files. A) All genes for which ESTs produced alignments to the reference genome of the related specie *B*. *mori*. B) Same as before considering only genes for which the ESTs coverage of data was above 0.125 RPKM. C) Assembled contigs that did not return signals of protein-coding capacities for which ESTs produced alignments.

Quality of the remaining ~13,000 contigs that did not fall into the previous attempts to identify their protein-coding potentialities were assessed to quality and accuracy accordingly to previously described in Materials and Methods. Fig. 1 summarizes our findings, suggesting that the sequencing of the 106,271 ESTs that generated the unannotated contigs occurred more frequently at the middle of the DNA strand towards to one of the ends (most probably the 3' end), and gave uneven profiling of the transcriptome assembly. This observed profile is very contrasting with the coverage data gathered from the ESTs that formed contig sequences that could be successfully placed on the genomic loci of a set of probable putative orthologous between SGB and the related specie *B*. *mori*. In this latter, the sequencing seems to occurs more frequently at the middle of the DNA strand and both ends are nearly equally represented. This analysis suggests that a bias occurred in the library prep and/or in the sequencing instrument that led to incomplete representation of this particular set of EST. Incomplete cDNA synthesis, inconsistent or poor fragmentation of the cDNA sample are known sources of biases that can be related to this observation [86]. These ESTs are almost certainly the product of poor quality sequencing for which the bioinformatics steps used previously the assembly process markedly reduce their coverage of data (mean depth of coverage 5.8x and contigs shorter than 1,200 bp). A similar discussion in a study of the transcriptome of midgut of *M*. *sexta* using 454 technology led to closely report in number of contigs that did not return informative similarity against known protein-coding sequences [28]. In this study the authors reported ~10,000 contigs in that condition. Accordingly to our analysis and similar findings in related literature we observe that we cannot offer an exhaustive picture of the SGB transcriptome. However we emphasize that sufficient caution was considered in our bioinformatics pipeline to establish a reliable set of sequences for downstream functional characterization at least at the same level of confidence observed in similar studies related to our sample.

**Fig 2:**
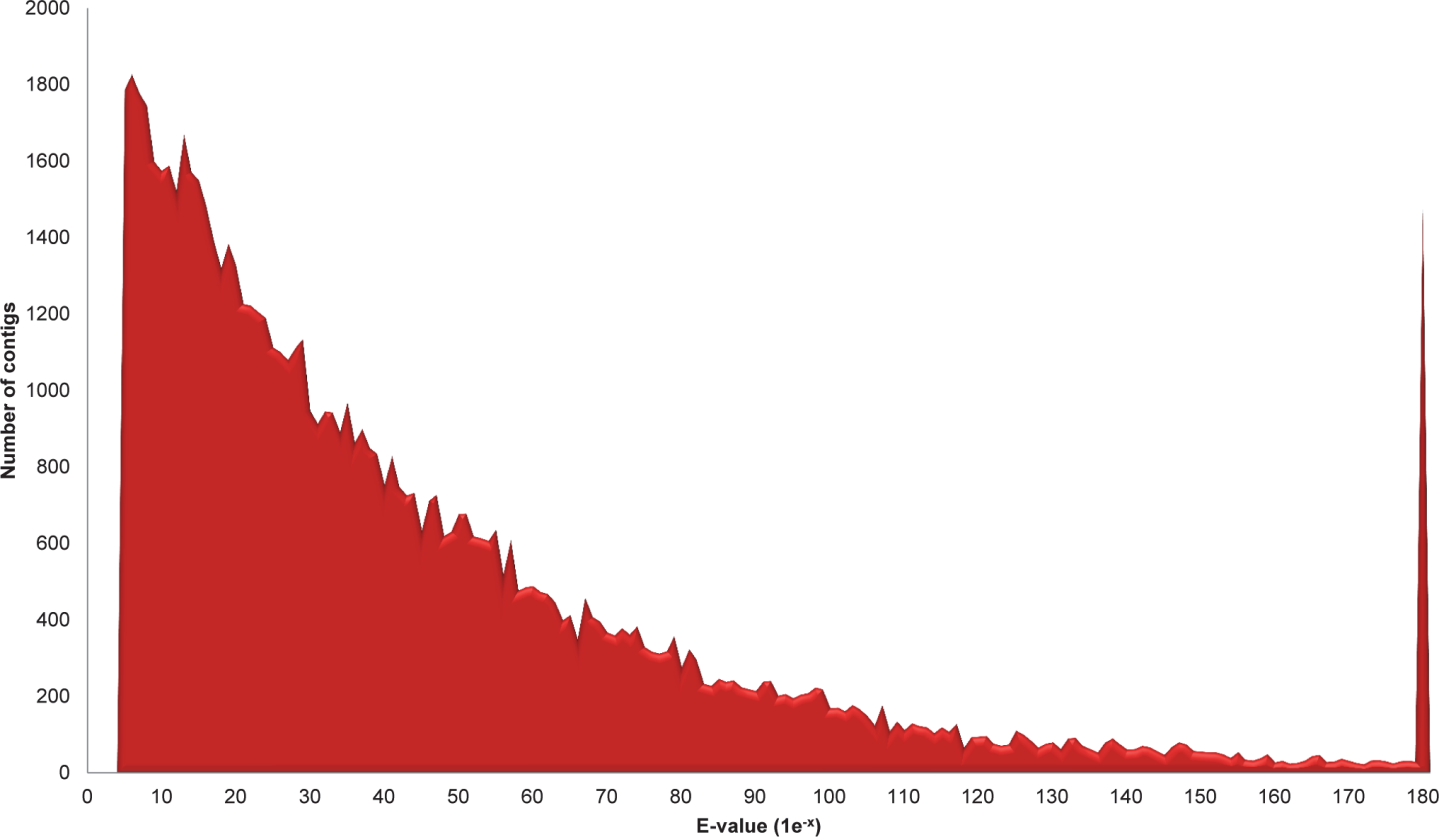
E-value distribution of the top BLASTx hits. Sequences with e-value equal to 0 are represented in the right peak. The cut-off used was 1e^-5^.

**Fig 3:**
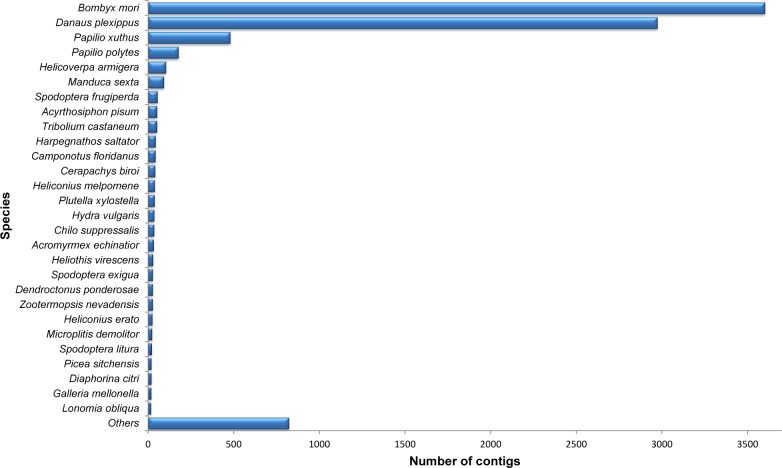
Species distribution of the top BLAST hits for each unique sequence. A higher similarity was observed with proteins from the lepidopteran *Bombyx mori*.

A high number of contigs with unknown functions were also observed on previously sequenced insect transcriptomes [20,87,88]. However, taking into account the inaccuracies in the sequencing, we cannot completely rule out that the lack of annotation can also suggests an important number of species-specific genes, which may be useful in several studies, particularly using RNAi strategies [24]. The BLASTx hits distribution, accordingly to the adopted e-value of 1e^-5^, is shown in Fig. 2. To determine the coverage of our library, we grouped the contigs accordingly to the most frequent species similarities. The highest percentage of sequence hits occurred with insect proteins, particularly Lepidoptera (30%), Hymenoptera (18%), Diptera (12%) and Coleoptera (7%). Though SGB is a lepidopteran, the high number of sequence similarities with dipterans and hymenopterans reflects mostly the influence of the large number of DNA sequences for these species in the GenBank. After comparing the top hits distribution, as expected, there was a higher percentage of similarity to protein sequences of lepidopterans (87%), especially *B*. *mori* (40%) and *Danaus plexippus* (33%) both with fully sequenced genomes (Fig. 3).

As the insects were collected directly from sugarcane fields and total RNA was isolated from whole body organisms, it was not possible to clean the content of the midgut, leaving the possibility for the presence of parasites and microorganisms, as well as plant tissues derived from the insect diet. Among our BLAST hits results, we observed a low number of contigs derived from species other than insects: *Branchiostoma floridae* (Lancelet or Amphioxus), *Hydra vulgaris* (Hydrozoan), *Saccoglossus kowalevskii* (Hemichordate), and *Picea sitchensis* (Seed plant). Although there is no known ecological relationship between the first three species and SGB, primarily because they have an aquatic lifestyle, a more detailed analysis of the contigs indicated that most of the sequences are associated with hypothetical proteins, possibly because the genome of those species have also been sequenced [89,90] and the lack of annotation is hampering the determination of protein function. The similarity of contigs with sequences of *P*. *sitchensis* could indicate contamination of our sample with plant tissues; however, all of the sequences were classified as unknown proteins. In fact, accordingly to the authors of the *P*. *sitchensis* sequencing project, there could be a contamination of their samples with insect cDNA since the plants were subjected to herbivory prior to RNA extraction and sequencing [91]. We searched our database for sequences of other plant species and found a few contigs with similarities to *Oryza sativa*, *Zea mays*, *Arabidopsis sp*. and *Vitis vinifera* but most of them code for transposon and retrotransposon proteins or proteins highly conserved between eukaryotes. No similarities were found after restricting the analysis of BLASTx (nr database) to *saccharum* sp sequences at the GenBank. Thus, there appears to be no significant influence of DNA contamination of SGB cDNA library.

### Gene Ontology and Function Classification

**Fig 4:**
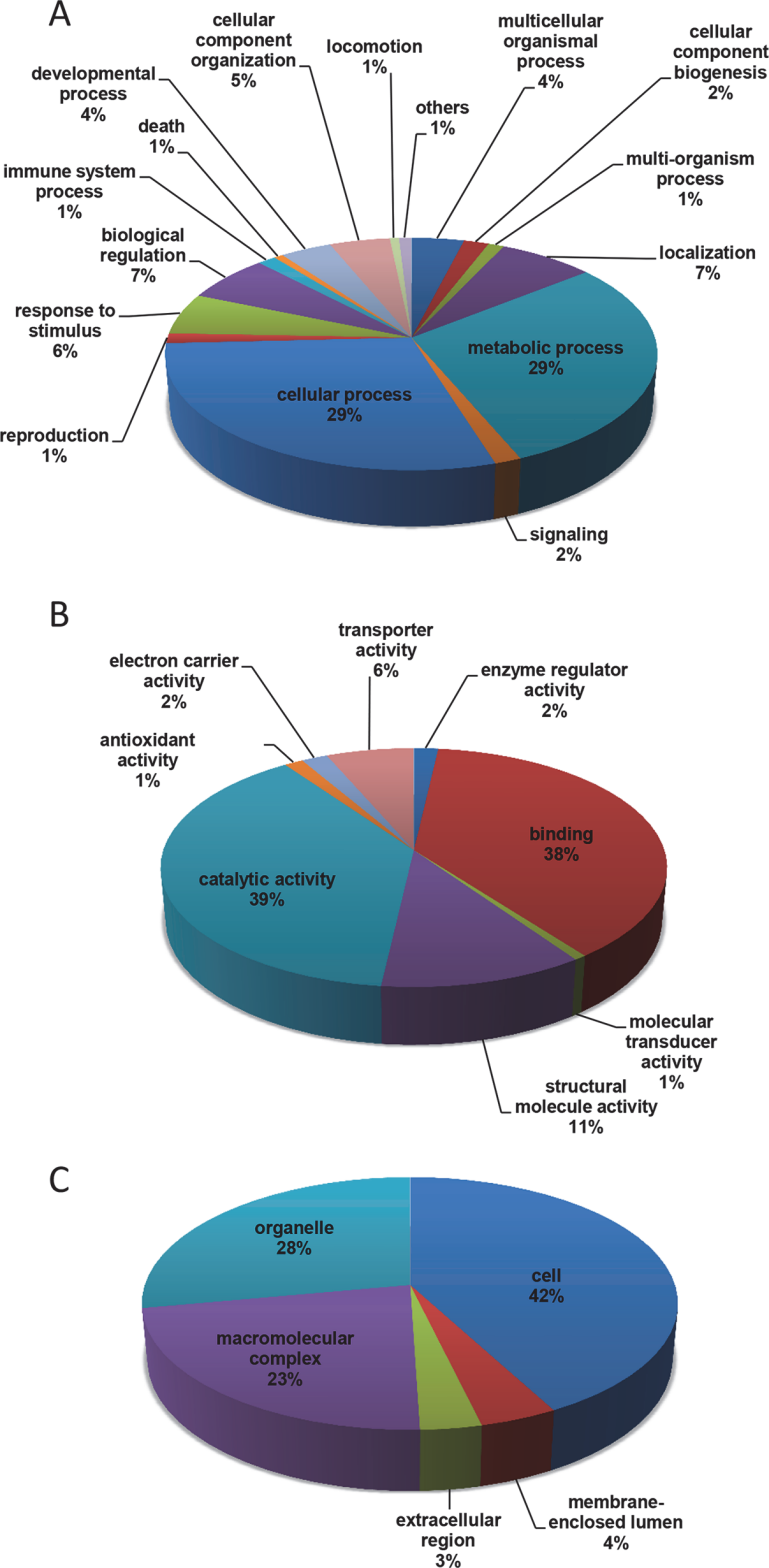
Gene Ontology (GO) assignments for SGB transcriptome. All contigs were classified on level 2 for **A**) Biological Process, **B**) Molecular Function and **C**) Cellular Component.

Gene ontology analysis (GO) was performed to classify the functions of the predicted proteins (Fig. 4). We observed a dominance of Biological Process GO terms for metabolic (29%) and cellular (29%) processes (Fig. 4A). For Molecular Function, it was observed a high percentage of terms for catalytic activity (39%) and binding (38%) (Fig. 4B). For Cellular Component, a high percentage of GO terms were predicted for cell components (42%) (Fig. 4C). The same pattern of GO classification was observed for other insect transcriptomes and confirmed that our database is representative and consistent with other reported data [92–95]. The InterPro database was used to obtain a more detailed classification of predicted proteins. Of almost 24,000 contigs, 16% presented InterPro entries (Table 1). The top 25 InterPro hits are shown in Table 2. The most frequent identified proteins were insect cuticle proteins (292 entries), NAD(P)-binding domain (264 entries) and cytochrome P450 (252 entries). Several contigs encoding putative digestive enzymes were also observed: peptidase S1/S6; chymotrypsin/Hap (218 entries), Serine/cysteine peptidase; trypsin-like (142 entries), carboxypeptidases (86 entries), lipases (84 entries) and peptidase S1A; chymotrypsin (79 entries) were the most frequent.

**Table 2:** Summary of top 25 protein domains found in the *Telchin licus licus* transcriptome.

InterPro	Frequency	Description
IPR000618	292	Insect cuticle protein
IPR016040	264	NAD(P)-binding domain
IPR001128	252	Cytochrome P450
IPR001254	218	Peptidase S1/S6, chymotrypsin/Hap
IPR002198	209	Short-chain dehydrogenase/reductase SDR
IPR015880	206	Zinc finger, C2H2-like
IPR007087	162	Zinc finger, C2H2-type
IPR002347	153	Glucose/ribitol dehydrogenase
IPR001680	144	WD40 repeat
IPR019781	143	WD40 repeat, subgroup
IPR009003	142	Serine/cysteine peptidase, trypsin-like
IPR000215	137	Protease inhibitor I4, serpin
IPR002557	132	Chitin binding protein, peritrophin-A
IPR000504	126	RNA recognition motif, RNP-1
IPR001395	105	Aldo/keto reductase
IPR002018	92	Carboxylesterase, type B
IPR000834	86	Peptidase M14, carboxypeptidase A
IPR000734	84	Lipase
IPR001251	83	Cellular retinaldehyde-binding/triple function, C-terminal
IPR000217	81	Tubulin
IPR001314	79	Peptidase S1A, chymotrypsin
IPR016196	73	Major facilitator superfamily, general substrate transporter
IPR005055	71	Insect pheromone-binding protein A10/OS-D
IPR012677	71	Nucleotide-binding, alpha-beta plait
IPR011046	69	WD40 repeat-like-containing domain

To increase our knowledge of proteins possibly expressed specifically in the SGB midgut, the EST library was compared with midgut sequences of *M*. *sexta* obtained from the InsectaCentral database. Out of 13,828 *M*. *sexta* sequences, 6473 hits were achieved above the cut-off (1x10^–3^); the most frequent InterPro hits are shown in S3 Table. Many of the contigs were classified as proteins for cell metabolism, digestion and detoxification. The presence of cuticle proteins among them is intriguing, although such characteristic has been observed in *Anopheles gambie* and *B*. *mori* and are most likely involved with immune defense response and midgut growth [96,97]. In addition, functional analyses of the BLAST hits were performed by grouping the contigs with a predicted function for digestive enzymes to estimate the number of unigenes and how many sequences from our library had no similarities to other known *M*. *sexta* genes (Table 3). Of the 120 contigs of serine protease transcripts identified in the SGB database, 96 presented BLAST hits against known midgut sequences, corresponding to 59 *M*. *sexta* unigenes. For aminopeptidase N, 16 contigs were found in our database and, interestingly, 18 contigs were obtained after cross-species similarities searches against *M*. *sexta* sequences using blat and gmap sequence alignment tools. The annotation of these two contigs could not be achieved previously because the sequences are too short and have no conserved domains, hampering to identify protein family groups.

**Table 3:** Digestive enzymes found in the *Telchin licus licus* transcriptome and their correspondence with *Manduca sexta* midgut enzymes.

Classification	InterPro	Total number of contigs	Number of contigs x *M*. *sexta* (unigenes)
Serine protease	IPR009003/IPR001314	120	96 (59)
Cystein protease	IPR015643	19	3 (2)
Carboxypeptidase	IPR000834	31	33 (18)
Aminopeptidase	IPR001930	16	18 (11)
Dipeptidase	IPR005320/IPR001548	9	9 (3)
α-amylase	IPR006047	15	9 (4)
α-glucosidase	IPR000322	4	5 (4)
β-glucosidase	IPR001360	12	13 (13)
β-galactosidase	IPR001944	3	3 (2)
Trehalase	IPR001661	2	2 (1)
Lipase	IPR006693/IPR000734/IPR013818/IPR002331	45	39 (16)

Additional functional, comparative and phylogenetic analysis of *de novo* transcriptome of SGB and *B*. *mori* genome was performed using the online tool TRAPID. Of all the groups shown in Table 4, many presented gene expansion or depletion among the gene families identified. In the serine protease group, the most notorious change occurred in family 1133_OG5_141149, in which there was an expansion of 9 genes, and in family 1133_OG5_130858, with a depletion of 8 genes. For the aminopeptidase group, there was an expansion of 1 gene in family 1136_OG5_129538. In general, there was expansion/depletion in almost all protein groups, except dipeptidase, α-glucosidase, β-glucosidase and trehalase (Table 4). Although more than one transcript could be part of the same gene, which could lead to an overrepresentation of the total number of genes, both species showed a close sequence count, indicating minimal influence by sequence mis-assembly. Changes in the number of genes are frequently found among eukaryotes and can be accounted for different mechanisms such as gene duplication, transposition and gene loss via the accumulation of mutations, leading to divergence of proteins function and adaptation. Even among closely related species, a large number of gene gain and loss can be observed. Depending on the biological functions of these genes it is reasonable to expect that such characteristic may influence the genotypic and phenotypic alterations observed among species [98]. The differences observed for SGB may explain the adaptation of the insect to a sugarcane based diet.

**Table 4:** Gene expansion/depletion analysis among *T*. *licus licus* and *B*. *mori* databases.

Classification	Gene family	Transcript count	
		*T*. *licus*	*B*. *mori*
Serine protease	1133_BGIBMGA002205	2	1
	1133_OG5_215701	4	2
	1133_OG5_149528	2	1
	1133_OG5_136800	3	1
	1133_BGIBMGA008883	2	1
	1133_OG5_174722	2	1
	1133_OG5_158561	3	1
	1133_BGIBMGA004487	2	1
	1133_OG5_158544	2	1
	1133_OG5_142367	2	1
	1133_OG5_141149	14	5
	1133_BGIBMGA004425	2	1
	1133_OG5_142493	3	1
	1133_BGIBMGA005172	2	1
	1133_OG5_152309	3	6
	1133_OG5_163947	1	6
	1133_OG5_130858	7	15
			
Cystein protease	1138_OG5_127800	3	1
	1138_OG5_126607	6	2
			
Carboxypeptidase	1134_BGIBMGA004799	3	1
	1134_OG5_128876	4	1
	1134_OG5_127925	1	2
			
Aminopeptidase	1136_OG5_129538	2	1
			
Alpha-amylase	1119_OG5_128640	7	3
			
Beta-galactosidase	1142_OG5_128163	2	1
			
Lipase	1135_OG5_130874	3	1
	1135_BGIBMGA004157	2	1
	1135_BGIBMGA011895	3	1
	1135_OG5_135981	2	4
	1135_OG5_150673	1	10

### Serine Proteases

Insects are adapted to almost all environments and can feed on a variety of nutrient sources such as grains, stems, cellulose, flesh and blood. Their diet serves as a primary source of fats, sugars and proteins that are digested by the action of several enzymes of the gut tract [36]. The major class of proteases found in the lepidopterans gut is the serine protease, particularly trypsin (EC 3.4.21.4) and chymotrypsin (EC 3.4.21.1); both characterized by the catalytic triad His 57, Asp 102, Ser 195 (bovine chymotrypsin numbering) [99]. Trypsin cleaves preferentially protein chains on the carboxyl side of basic amino acids, such as arginine and lysine. Conversely, chymotrypsin cleaves protein chains on the carboxyl side of aromatic amino acids, such as phenylalanine and tyrosine [36]. The specificity of serine proteases is determined by a (S1) binding pocket that interacts with the side chains of amino acids at the P1-P1’ site of the substrate [100]. In trypsin-like proteases the main residues that form the S1 pocket are Asp 189, Gly 216 and Gly 226. Chymotrypsin-like proteases usually contain the residues Gly/Ser 189, Gly 216 and Gly 226 [101].

In the SGB database several contigs for serine proteases were identified by InterPro analyses. Searching for those contigs presenting complete protein coding sequences, five contigs for trypsin-like and four chymotrypsin-like proteases were found and later confirmed by BLAST search against NCBI nr database. According to the MEROPS peptidase database (http://merops.sanger.ac.uk), all the sequences were classified as belonging to the SA1 family and many preserved all the characteristics of digestive serine proteases.

**Fig 5:**
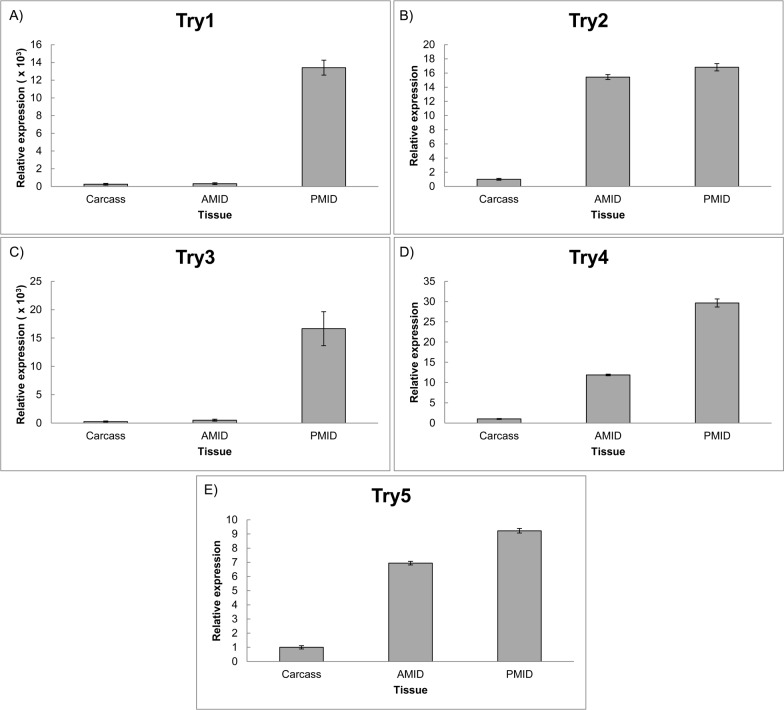
Real time PCR of trypsin-like genes in three different tissues of SGB. A) Trypsin 1, B) Trypsin 2, C) Trypsin 3, D) Trypsin 4 and E) Trypsin 5. AMID (Anterior midgut). PMID (Posterior midgut). Each bar represents the relative expression in comparison to the tissue that had the smaller expression value, arbitrarily designated as 1. Standard errors of technical triplicate are shown.

**Fig 6:**
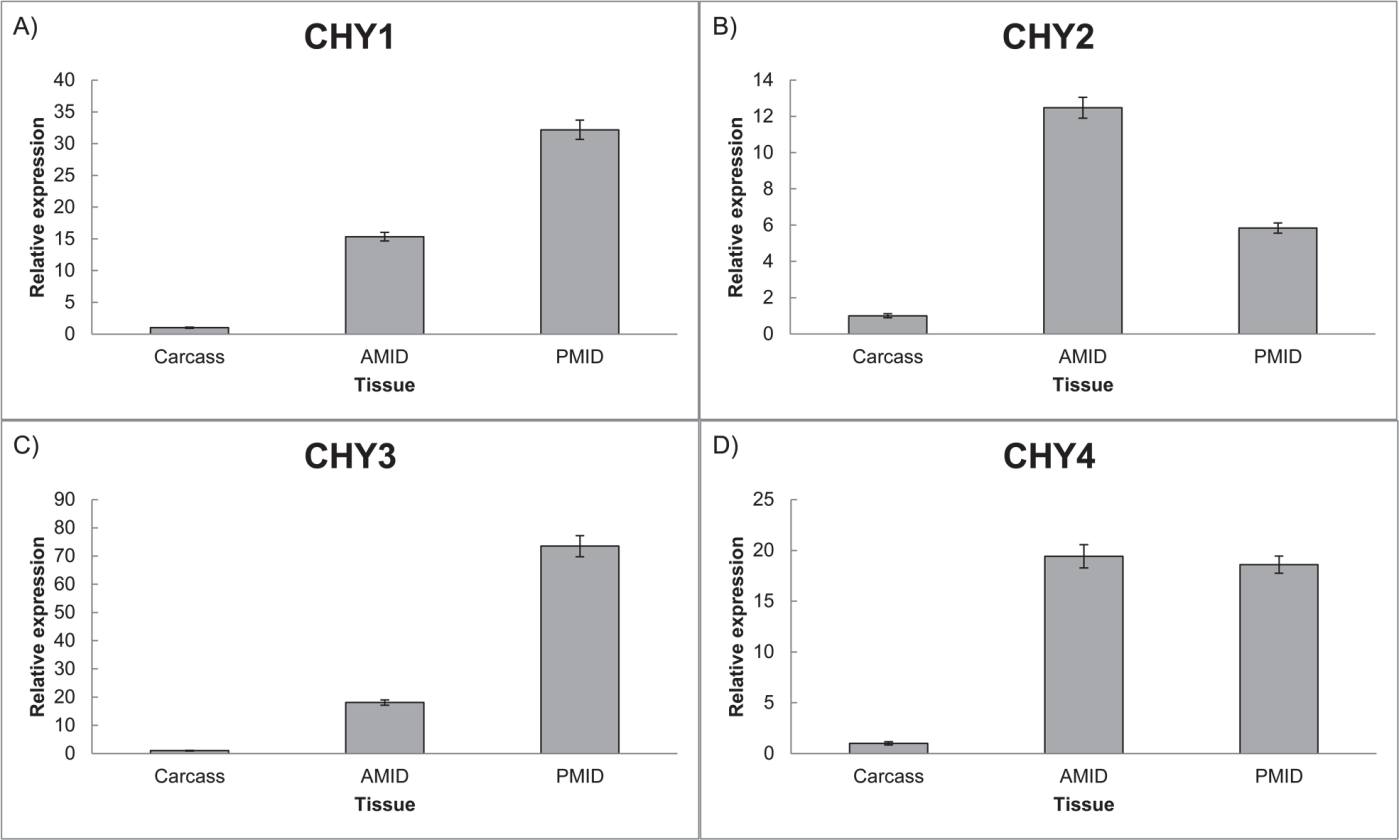
Real time PCR of chymotrypsin-like genes in three different tissues of SGB. A) Chymotrypsin 1, B) Chymotrypsin 2, C) Chymotrypsin 3 and D) Chymotrypsin 4. AMID (Anterior midgut). PMID (Posterior midgut). Each bar represents the relative expression in comparison to the tissue that had the smaller expression value, arbitrarily designated as 1. Standard errors of technical triplicate are shown.

The sequences have a 5’ UTR and 3’ UTR regions and an open reading frame (ORF) of approximately 800 bp. The trypsin-like transcripts coded for predicted proteins of 259–306 amino acids with predicted isoelectric points ranging from 5.48 to 8.70 and theoretical molecular weights of 26 to 33 kDa. All the genes presented higher transcript levels in the midgut tissues, compared to the carcass (Fig. 5 A-E). The same characteristic was observed with other insect serine proteases, indicating that the SGB genes could be part of the intestinal homogenate and participate on digestion of proteins [102]. The chymotrypsin-like transcripts coded for predicted proteins of 279–299 amino acids with isoelectric points ranging from 6.67 to 8.27 and molecular weights of 29 to 32.8 kDa (S4 Table). Likewise, the chymotrypsin genes presented higher expression in the midgut (Fig. 6 A-D). The characterization of several trypsin- and chymotrypsin-like *Ostrinia nubialis* cDNAs revealed that insect digestive proteases could possess similar characteristics [103].

**Fig 7:**
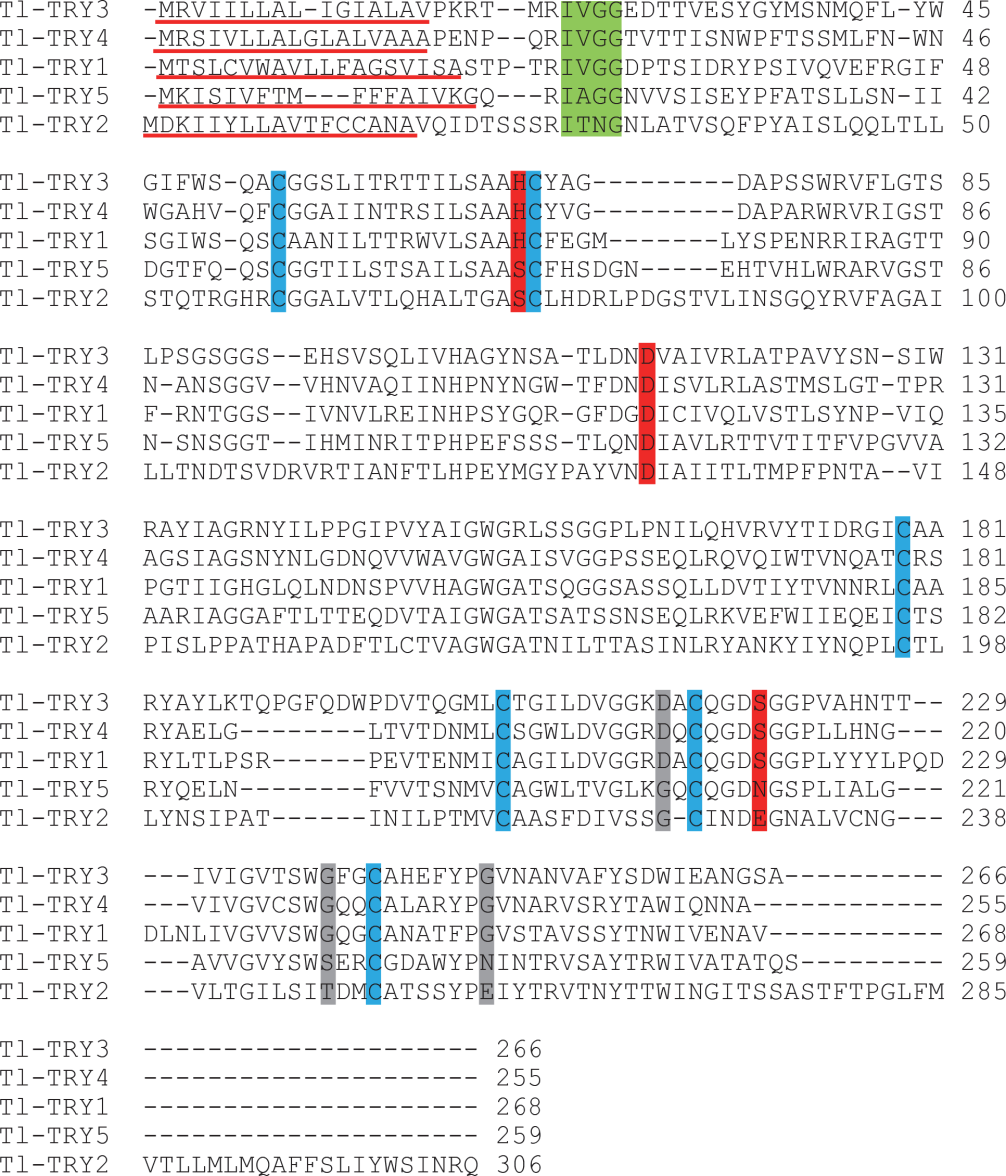
ClustalW2 alignment of *T*. *licus licus* trypsin-like proteins. Many sequences preserved the characteristics of digestive serine proteases. The signal peptide is shown with a red underline. The cleavage site is shown in a green box. Red boxes indicate the active site residues. Grey boxes indicate the substrate binding region and cysteines that are possibly involved with disulfide bonds are shown in blue.

Sequence alignment was carried out to identify conserved sites. The trypsin-like transcripts were named Tl-TRY1—Tl-TRY5 and contain an N-terminal signal peptide. A conserved IXGG (where X stands for any amino acid) propeptide-processing site was observed on all sequences but not in Tl-TRY2, suggesting that this molecule could remain in the organism as a zymogen. The catalytic triad was conserved on sequences 1, 3 and 4 as well as the binding pocket site. For sequence Tl-TRY2, His74 was replaced by Ser, Ser230 was replaced by Glu and the binding pocket contained substitutions in all residues. Sequence Tl-TRY5 conserved only Asp114, which is part of the catalytic site (Fig. 7). As a mechanism for avoiding the effects of PIs, insects change the expression of many proteases, leading to activate many digestive enzymes or to produce insensitive proteins with sequence variations that helps to prevent the binding of PIs [104,105], explaining the high number of trypsin genes in the genome and transcriptome databases. Another important characteristic observed in the insect physiology is the expression of proteases with mutations at the active site, which could indicate that these molecules are inactive [106]. Notwithstanding, such molecules have been identified in several insects, and many of them classified as serine protease homologs (SPH) that are possibly involved in innate immune response [107,108]. The amino acid substitutions observed on sequences 2, and 5 are easily found in serine protease sequences at the GenBank (S1 Fig. and S2 Fig.) and most likely indicate that such mechanism could be conserved among different insect species.

**Fig 8:**
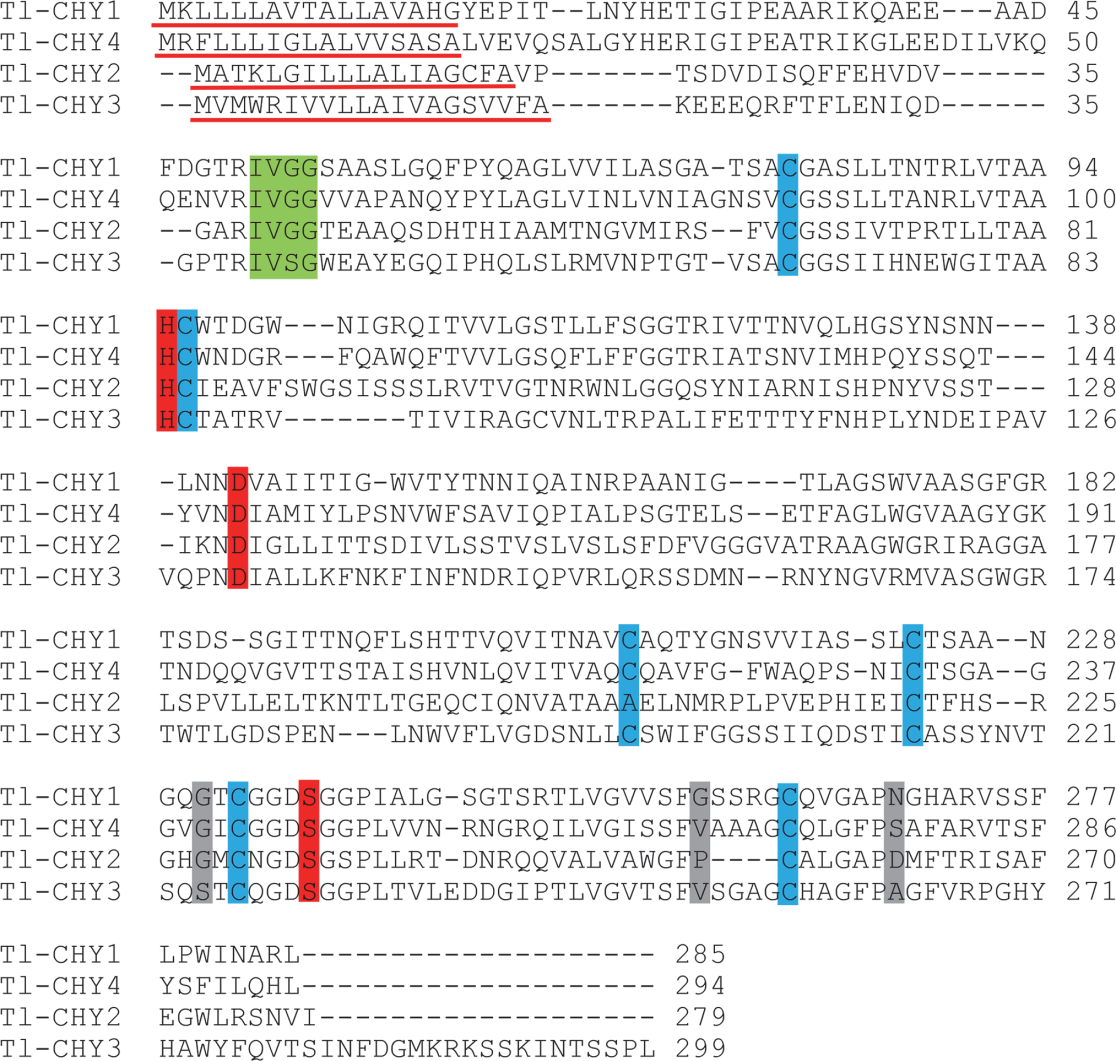
ClustalW2 alignment of *T*. *licus licus* chymotrypsin-like proteins. Many sequences preserved the characteristics of digestive serine proteases. The signal peptide is shown with a red underline. The cleavage site is shown in green box. Red boxes indicate the active site residues. Grey boxes indicate the substrate binding region and cysteines that are possibly involved with disulfide bonds are shown in blue.

Chymotrypsin-like transcripts were named Tl-CHY1—Tl-CHY4 and they preserved the N-terminal signal peptide. A conserved IXGG site is observed in all sequences except Tl-CHY3. The catalytic triad was conserved in all sequences. Substrate binding sites contained amino acid substitutions at different positions (Fig. 8). The differences in the binding pocket sites indicate that these enzymes have diverse activities because chymotrypsin sequences show more variations at the S1 pocket, which could increase protein flexibility and result in differential substrate recognition [41]. Although SGB serine protease activity has yet to be experimentally investigated, the identification and characterization of different genes will help researches to understand the enzymatic arsenal used by the insect to process the nutritive content of a sugarcane-based diet and to study protease involvement in the Cry toxin activation/deactivation mechanism. Such information will be important to guide the development of alternative strategies for pest control, such as genetic transformation of Elite sugarcane events by expressing Cry toxins or PIs, targeting specifically the activity of digestive enzymes [109,110].

### Aminopeptidases

Midgut aminopeptidases N (APNs—EC 3.4.11.2) are enzymes that participate in the digestion of proteins by cleaving neutral amino acids from the N-terminus of polypeptides [36]. APNs are classified as belonging to the M1 family and have a zinc binding motif HEXXH (where X stands for any amino acid), followed by a conserved glutamic acid 24-amino acids downstream from the first histidine. While the histidines and the last glutamic acid form the zinc binding region, the first glutamic acid participate in the catalytic process [111]. A highly conserved GAMEN motif is also found upstream from the zinc binding motif and is believed to be a part of the active site [112]. In insects, APNs have a cleavable N-terminal signal peptide that directs the protein to the outer surface of the cell membrane where they are attached through a glycosylphosphatidylinositol (GPI) anchor located at the C-terminus [113].

Several lepidopteran APNs have been experimentally shown to bind different classes of Bt δ-endotoxins [31]. According to the most recent pore formation model, Cry1A toxins, once activated in the midgut of a susceptible insect, participate in a series of binding events with protein receptors present in the intestinal epithelium. In *M*. *sexta*, where the mode of action is better characterized, the first interaction consists of weak binding of a monomeric toxin to an APN or ALP receptor, allowing its recognition by Cadherin receptors. The protein-protein interactions induce an oligomer formation of Cry molecules, which binds again to the APN and is introduced into the plasma membrane forming a pore that causes osmotic lysis [114]. Other lepidopteran APNs were shown to participate in the development of resistance to insecticides [115] suggesting that such molecules are important targets for insect population management.

**Fig 9:**
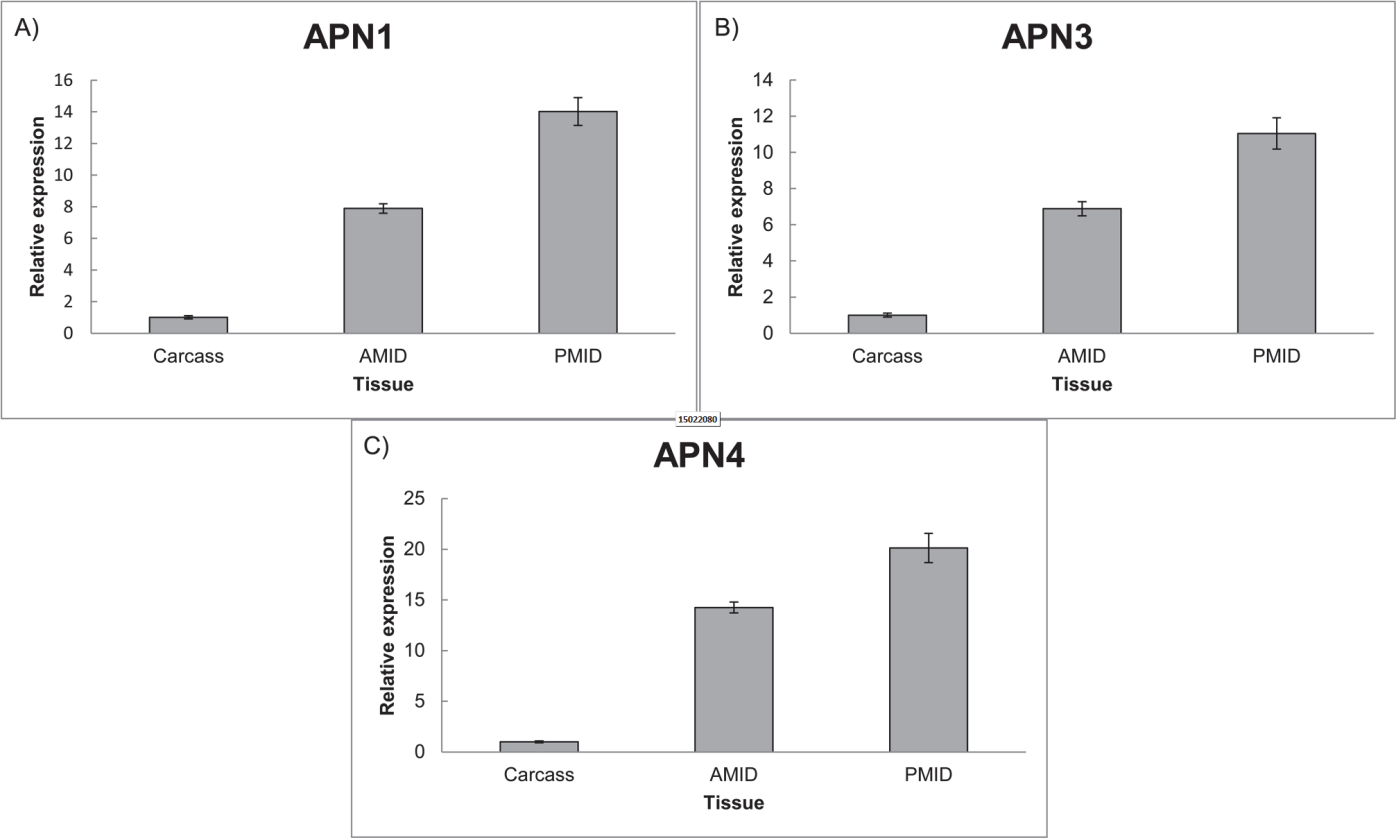
Real time PCR of APN genes in three different tissues of SGB. A) Aminopeptidase 1, B) Aminopeptidase 2 and C) Aminopeptidase 3. AMID (Anterior midgut). PMID (Posterior midgut). Each bar represents the relative expression in comparison to the tissue that had the smaller expression value, arbitrarily designated as 1. Standard errors of technical triplicate are shown.

In the SGB database, a total of 18 APN contigs were identified after a similarity search against *M*. *sexta* cDNA sequences. Two contigs are actually two complete protein coding sequences and a third contig lacked the N-terminal region that was solved by 5’-RACE (S3 Fig.). Real time PCR experiments showed that APN transcript levels were higher in midgut tissues than the carcass (Fig. 9 A-C).The protein sequences were named TlAPN1, TlAPN3 and TlAPN4, according to the phylogenetic relationships of lepidopteran APNs. All sequences coded for proteins of approximately 1000 amino acids. A glycosylphosphatidylinositol anchoring signal, as well as an N-terminal signal peptide of 20 amino acids were predicted for all proteins. Sequence alignment confirmed that the HEXXH(X)_18_E and GAMEN motifs are also conserved (S4 Fig.). The percentage of identity among the APN proteins ranged from 34% to 44% (S5 Table).

Glycosylation sites were predicted for different amino acid residues over the entire protein sequences. Four N-glycosylations and four O-glycosylations were predicted for TlAPN1, whereas six N-glycosylations and 18 O-glycosylations where predicted for TlAPN3. Moreover, six N-glycosylations and two O-glycosylations were predicted for TlAPN4 (S6 Table). It has been shown in previous reports that Cry1Ac toxins are capable of interacting with N-acetyl-galactosamine (GalNAc). In fact, competitive assays demonstrated that GalNAc binding to the toxin impairs the interaction with the plasma membrane receptors [116], which could reflect on a reduced activity. According to Sangadala and coworkers (2001) [117], there is a proportion of 4% carbohydrate in GPI cleaved *Manduca sexta* aminopeptidase 1 (MsAPN1), which presents a molar ratio of approximately 6: 10: 7: 3 for, respectively, GalNAc/GlcNAc/Man/Fuc. Glycosylation prediction sites indicate the presence of four possible N-glycosylations and 13 O-glycosylations in MsAPN1 [118]. A more detailed analysis of the N-linked oligosaccharides of MsAPN1 through mass spectrometry has revealed that 3 of the 4 N-glycosylations are occupied with highly fucosylated N-glycans (Hex_3_HexNAc_3_Fuc_3_), while the remaining site is occupied by a paucimannosidic N-glycan (Man_3_GlcNAc_2_) [119]. No GalNAc was observed in any of the major N-glycans on MsAPN1, suggesting that 5 or 6 of the O-glycosylation sites are occupied with simple (GalNAc-peptide) type O-glycans [118,119]. It is believed that the presence of GalNAc in the C-terminus and its proximity to the plasma membrane could increase the affinity and specificity of binding to Cry1Ac toxin [120].

**Fig 10:**
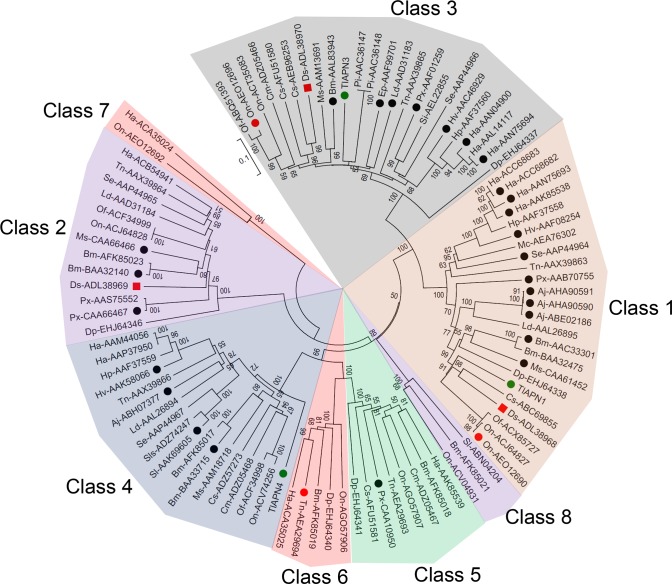
Phylogenetic analysis of representative lepidopteran APNs. The proteins of *T*. *licus licus* are shown in green. Black spots indicate the APNs that have been reported as putative Cry toxin receptors. Red spots indicate the APNs which gene expression changed after Bt infection. Red squares indicate the APNs which silencing induced resistance of the insect to Cry1Ab. GenBank accession number is shown for each protein. Species abbreviations: Ha, *Helicoverpa armigera*; Hp, *Helicoverpa punctigera*; Hv, *Heliothis virescens*; Px, *Plutella xylostella*; Se, *Spodoptera exigua*; Tn, *Trichoplusia n*i; Bm, *Bombyx mori*; Ms, *Manduca sexta*; Ld, *Lymantria dispar*; Ep, *Ephiphyas postvittana*; Pi, *Plodia interpunctella*; Sl, *Spodoptera litura*; Cs, *Chilo suppressalis*; Sls, *Spodoptera litoralis*; Cm, *Cnaphalocrocis medinalis*; Ds, *Diatraea saccharalis*; Si, *Sesamia inferens*; Dp, *Danaus plexippus*; Os, *Ostrinia nubilalis*; Mc, *Mamestra configurata*; Aj, *Achaea janata*; Of, *Ostrinia furnacalis*.

To better characterize SGB APNs, a phylogenetic analysis was performed to discern evolutionary relationships among representative APNs of several lepidopteran species. The sequences were fully distributed among eight phylogenetic classes, unlike the five classes previously identified when there were not much APN sequences at the GenBank [121]. Protein sequences of SGB were grouped in classes, 1, 3 and 4 (Fig. 10). Many of these proteins were reported as possible participants in the Cry mechanism of action, either by direct interaction with the toxin or by changing its expression levels in response to Bt infection. More than one APN for the same species was grouped among the eight classes, which could indicate that the protein belongs to a group of duplicated APN genes [122] or was derived from different tissues rather than just the midgut [123]. Class 1 presented proteins that were previously reported as Cry toxin receptors [31] as well as proteins identified in recent publications. Three *Helicoverpa armigera* proteins were included in the analysis. Two of them (accession numbers: ACC68682 and ACC68683) are associated with the accumulation of mutations that led to the development of resistance to Cry1Ac [124] and the third sequence (accession number: AAN75693) represents a recombinantly expressed protein that interacts differently with Cry1A toxins [125]. This group also included the *Ostrinia nubialis* sequence (accession number: AEO12690), which gene expression was substantially decreased in Cry-resistant insects [126] and *Diatraea saccharalis* APN1, in which silencing trough RNAi increased insect survival to Cry1Ab [59]. Class 2 included a *D*. *saccharalis* APN2 that was involved with survival of the insect to Cry1Ab survival. Class 3 presented a *D*. *saccharalis* APN3 as well as an *O*. *nubialis* APN (AEO12696), both of which were involved with Cry1Ab resistance. Class 4 added sequences that had not been observed in previously reported phylogenetic trees, including *Achaea Janata* APN, which binds to Cry1A toxins [127]. Class 5 was composed of eight proteins, but only one is known to bind to Cry toxins [128]. Class 6 was a group first observed by Crava and coworkers (2010) and included APNs of two insect species [129]. In this work, the analysis extended the number of APN proteins to six species, including a *Trichoplusia ni* APN6 that was significantly up-regulated in greenhouse-evolved resistant insects [60]. Classes 7 and 8 are composed of a few insect species but lacked any reported Cry toxin binding information. The features observed between SGB and other lepidopteran APNs, associated with the susceptibility of the insect to Cry toxins, indicate that some of these proteins could act as toxin receptors, raising the possibility of using such information to develop Cry toxins with increased activity towards specific targets.

## Conclusions

In this work we report on the construction of a database of sugarcane giant borer (*T*. *licus licus*) by pyrosequencing of whole body mRNAs from several life stages. Before the data here presented, only 60 mitochondrial DNA sequences were deposited at the GenBank for this subspecies. As such, we believe our reported data will contribute significantly to advance future research on this organism. We have started characterizing many serine proteases and aminopeptidases, aiming specifically to study midgut enzymes that could be used in pest management assays. Next, we will characterize the activity of the serine proteases and determine the pattern of expression of these genes during the insect life cycle. To unravel the role of Cry toxin susceptibility we identified putative receptors that will be tested for their ability to bind to several Cry toxins. In addition, we intend to identify and validate genes through RNAi, a study which also will contribute to the better understanding of the insect’s developmental biology.

## Supporting Information

S1 Fig ClustalW2 alignment of SGB trypsin-like Tl-TRY2 contig. BLASTp results show that sequence variation on the substrate binding site is frequently found at the GenBank. The cleavage site is shown in green. Red boxes indicate the active site. Gray boxes indicate the substrate binding region and blue boxes show the cysteins that are most likely involved with disulfide bonds. Abbreviations: Mc, *Mamestra configurata*; Ha, *Helicoverpa armigera*; Dp, *Danaus plexippus*. GenBank accession numbers are indicated.
(TIFF)

S2 Fig ClustalW2 alignment of SGB trypsin-like Tl-TRY5 contig. BLASTp results show that sequence variation on the substrate binding site is frequently found at the GenBank. The cleavage site is shown in green. Red boxes indicate the active site. Gray boxes indicate the substrate binding region and blue boxes show the cysteins that are most likely involved with disulfide bonds. Abbreviations: On, *Ostrinia nubialis*; Cs, *Chilo suppressalis*; Ms, *Manduca sexta* Of, *Ostrinia furnacalis*; Dp, *Danaus plexippus*. GenBank accession numbers are indicated.
(TIFF)

S3 Fig Agarose gel showing the 750 bp DNA fragment that was sequenced to complete the TlAPN1 N-terminus region. 1) 1Kb DNA Plus ladder (Invitrogen Life Sciences). 2) PCR product.
(TIF)

S4 Fig ClustalW2 alignment of *Telchin licus licus* APNs. Signal peptide is indicated with a red underline. Blue and green boxes show the active site of the protein. Red boxes indicate the predicted GPI anchoring site.
(TIF)

S1 Table Primer sequences for amplification of TlAPN1 N-terminus. (DOCX)

S2 Table Primer sequences for qPCR of protease contigs. (DOCX)

S3 Table Top protein domains found in *Telchin licus licus* transcriptome after BLASTx against *Manduca sexta* midgut proteins. (DOCX)

S4 Table Theoretical physico-chemical parameters of serine proteases identified in SGB transcriptome. (DOCX)

S5 Table Amino acid sequence identity among SGB APNs. (DOCX)

S6 Table Glycosylation sites predicted for SGB APNS. (DOCX)
