# Correction: Sugarcane Giant Borer Transcriptome Analysis and Identification of Genes Related to Digestion

**DOI:** 10.1371/journal.pone.0123836

**Published:** 2015-04-02

**Authors:** The PLOS ONE Staff

The fifth author’s name is spelled incorrectly. The correct name is: José Dijair Antonino de Souza Júnior. The correct citation is: de Assis Fonseca FC, Firmino AAP, de Macedo LLP, Coelho RR, de Souza Júnior JDA, Silva-Junior O, et al. (2015) Sugarcane Giant Borer Transcriptome Analysis and Identification of Genes Related to Digestion. PLoS ONE 10(2): e0118231. doi:10.1371/journal.pone.0118231
