# Supplementary figures and images for: Sugarcane Giant Borer Transcriptome Analysis and Identification of Genes Related to Digestion
Source: PLoS One. 2015 Feb 23;10(2):e0118231. doi: 10.1371/journal.pone.0118231 (PMC4338194; doi:10.1371/journal.pone.0118231)

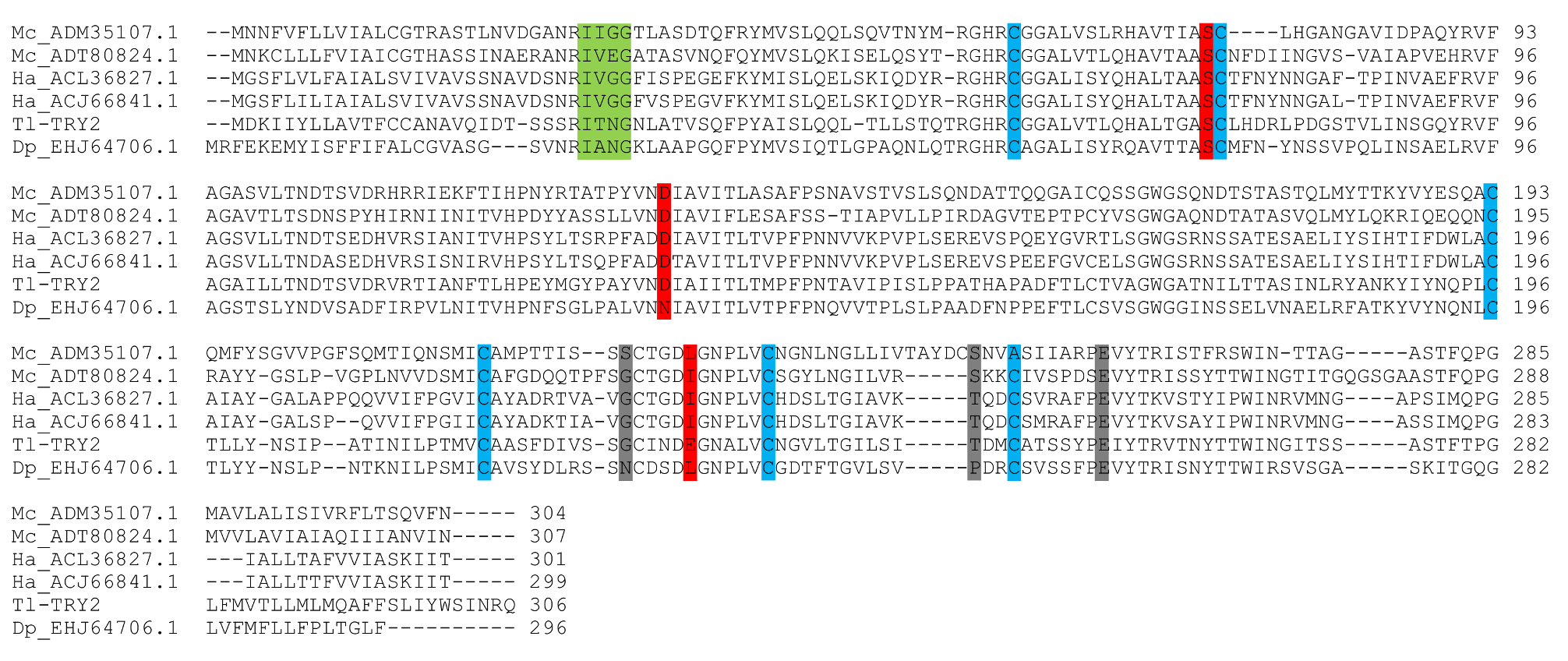

Supplement: S1 Fig — BLASTp results show that sequence variation on the substrate binding site is frequently found at the GenBank. The cleavage site is shown in green. Red boxes indicate the active site. Gray boxes indicate the substrate binding region and blue boxes show the cysteins that are most likely involved with disulfide bonds. Abbreviations: Mc, Mamestra configurata; Ha, Helicoverpa armigera; Dp, Danaus plexippus. GenBank accession numbers are indicated. (TIFF) [file pone.0118231.s001.tiff]

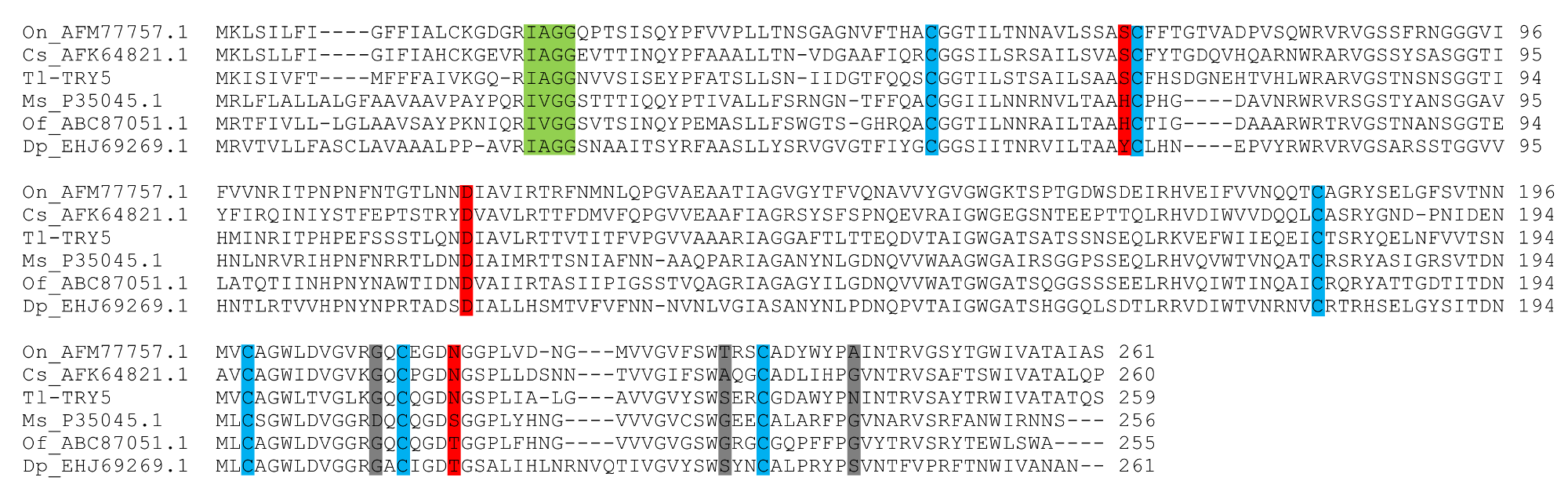

Supplement: S2 Fig — BLASTp results show that sequence variation on the substrate binding site is frequently found at the GenBank. The cleavage site is shown in green. Red boxes indicate the active site. Gray boxes indicate the substrate binding region and blue boxes show the cysteins that are most likely involved with disulfide bonds. Abbreviations: On, Ostrinia nubialis; Cs, Chilo suppressalis; Ms, Manduca sexta Of, Ostrinia furnacalis; Dp, Danaus plexippus. GenBank accession numbers are indicated. (TIFF) [file pone.0118231.s002.tiff]

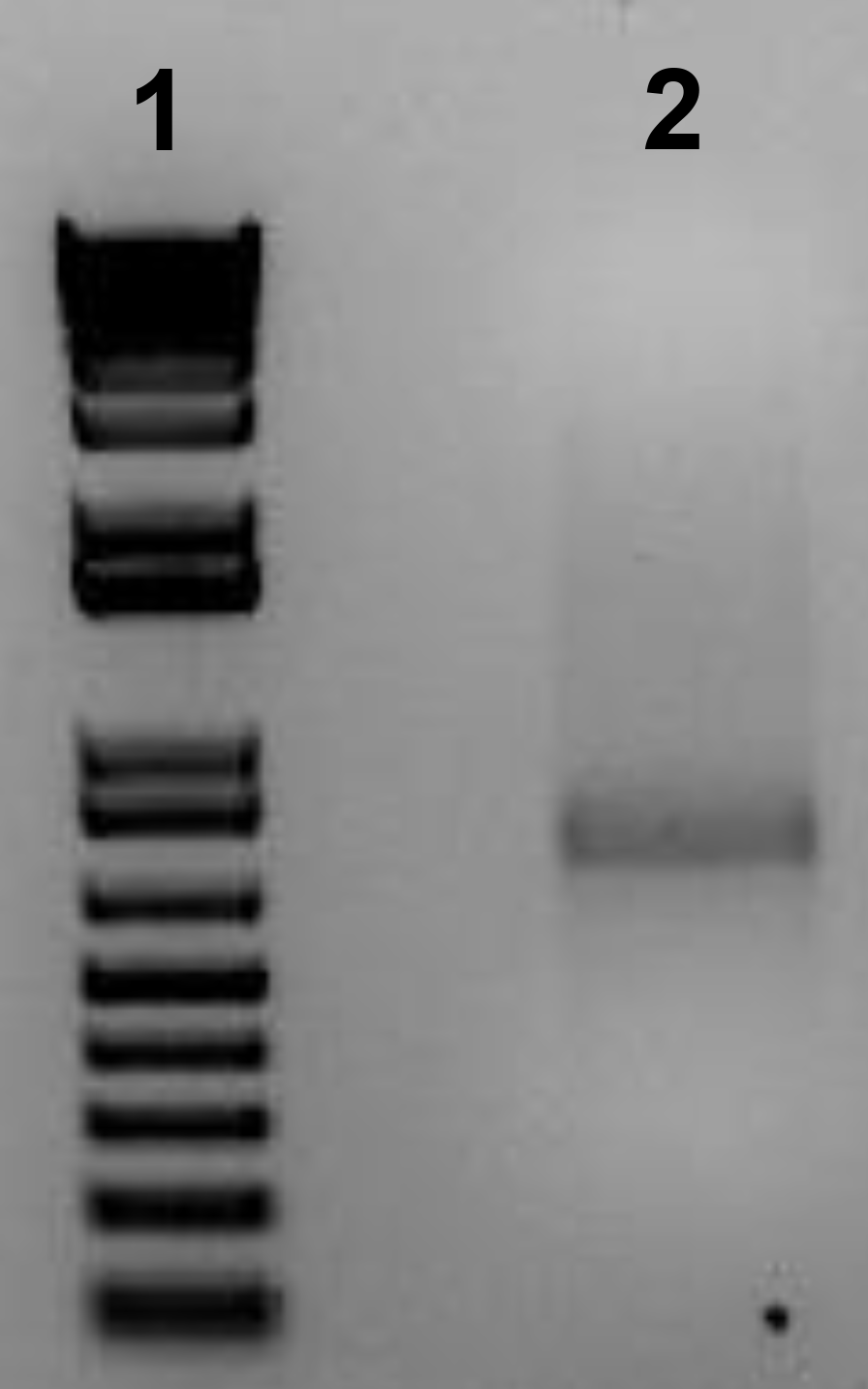

Supplement: S3 Fig — 1) 1Kb DNA Plus ladder (Invitrogen Life Sciences). 2) PCR product. (TIF) [file pone.0118231.s003.tif]

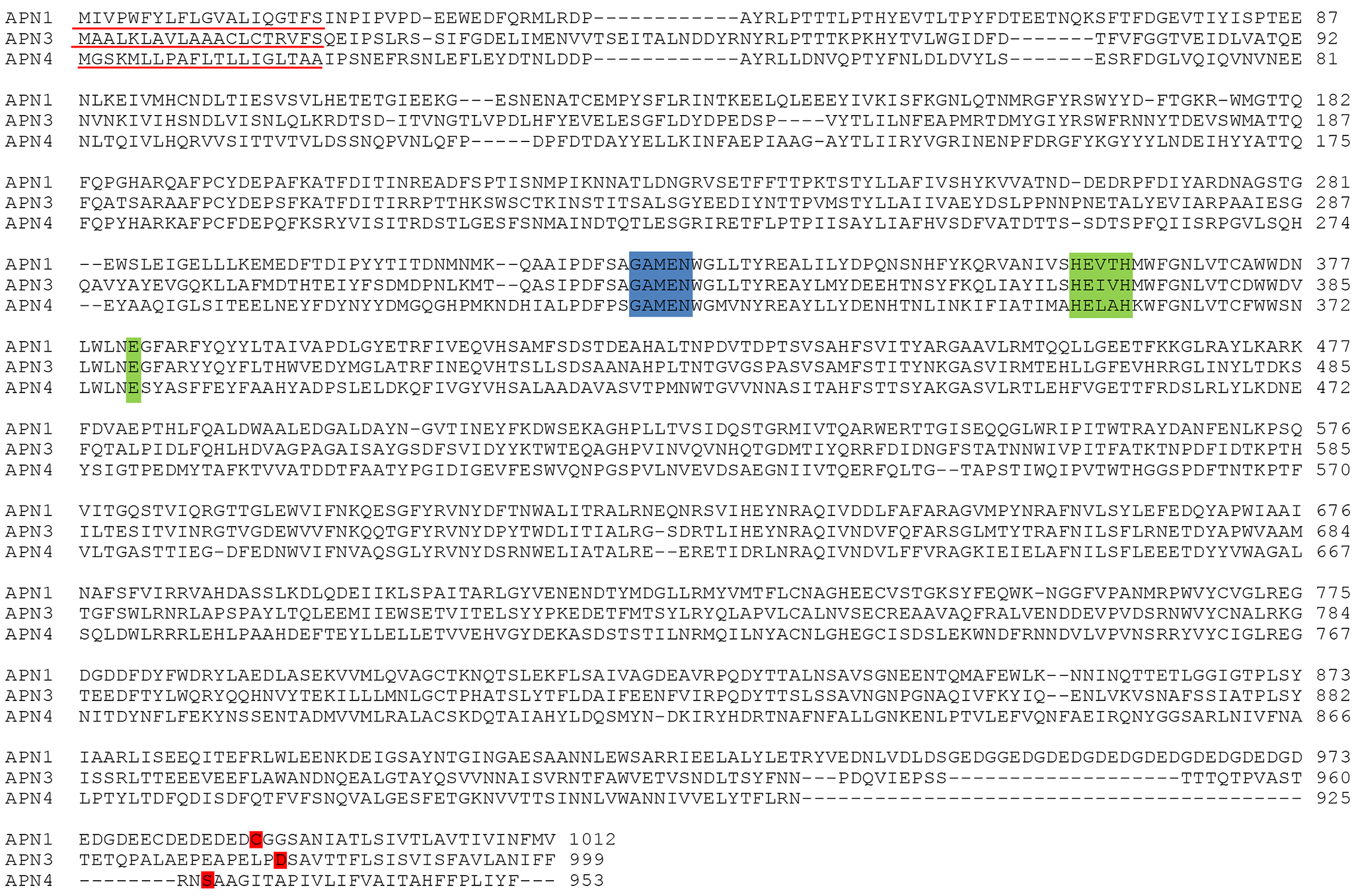

Supplement: S4 Fig — Signal peptide is indicated with a red underline. Blue and green boxes show the active site of the protein. Red boxes indicate the predicted GPI anchoring site. (TIF) [file pone.0118231.s004.tif]
