# Supplementary material for: Sugarcane Giant Borer Transcriptome Analysis and Identification of Genes Related to Digestion
Source: PLoS One. 2015 Feb 23;10(2):e0118231. doi: 10.1371/journal.pone.0118231 (PMC4338194; doi:10.1371/journal.pone.0118231)
Supplement: S1 Table — (DOCX) [file pone.0118231.s005.docx]

| **Primer** | **Sequence 5' - 3'** |
| --- | --- |
| gene specific 1 | AACGCGCTGCTTGTAGAAAT |
| gene specific 2 | GCCGCCTGTTTCATATTCAT |
| gene specific 3 | ATGGTGGGACTGAAATCTGC |
| oligo-dT adapter | GAATTCACGCGTCGACTAGTAGCATATGTACTTTTTTTTTTTTTTTTTTTTTTTTTTTTTTVN |
| anchor primer | GGGGACCACTTTGTACAAGAAAGCTGGGTTCCGAATTCACGCGTCGACTAGTAGCA |

**S1 Table.** **Primer sequences for amplification of TlAPN1 N-terminus.**
