# Supplementary material for: Sugarcane Giant Borer Transcriptome Analysis and Identification of Genes Related to Digestion
Source: PLoS One. 2015 Feb 23;10(2):e0118231. doi: 10.1371/journal.pone.0118231 (PMC4338194; doi:10.1371/journal.pone.0118231)
Supplement: S2 Table — (DOCX) [file pone.0118231.s006.docx]

| **Primer** | **Sequence 5' - 3'** |
| --- | --- |
| TlTRY1fw1 | TGCAGGTAGCGTGATTTCTG |
| TlTRY1rv1 | TACCCCTGAATTCCACTTGC |
| TlTRY2fw1 | TTCCTGCTTGCATGACAGAC |
| TlTRY2rv1 | TGTCCACCGAAGTGTCGTTA |
| TlTRY3fw1 | CCTCCTGGCATTCCTGTTTA |
| TlTRY3rv1 | AGATACCGCGGTCAATGGTA |
| TlTRY4fw1 | GGTCTCACTGTCACCGACAA |
| TlTRY4rv1 | CATACACCGACGATGACACC |
| TlTRY5fw1 | CCGTCGGTCTTAAAGGACAA |
| TlTRY5rv1 | ACCAGGCATCACCACATCTT |
| TlCHY1fw1 | CGAATAACATCCAGGCCATC |
| TlCHY1rv1 | TGATGCCAGAGGAATCACTG |
| TlCHY2fw1 | GTGATCACACGCACATAGCC |
| TlCHY2rv1 | CAATGCGCAGCAGTAAGAAG |
| TlCHY3fw1 | AACGTTACCAGCCAATCGAC |
| TlCHY3rv1 | GCCCGAAACGAAAGATGTAA |
| TlCHY4fw1 | GTCCGAATTGTTGGAGGTGT |
| TlCHY4rv1 | CGCAGACTGAGTTACCAGCA |
| Tl_APN1_qPCRfw1 | AAGATCTGGCAAGCGAGAAA |
| Tl_APN1_qPCRrv1 | TCTGACTGCCTCATCACCAG |
| Tl_APN3_qPCRfw1 | GCTTATGAAGTGGGCCAAAA |
| Tl_APN3_qPCRrv1 | TGAAAAATCGGGAATGGAAG |
| Tl_APN4_qPCRfw1 | CTTCGATGAGCCACAGTTCA |
| Tl_APN4_qPCRrv1 | CGAGGGTTTGTGTGTCATTG |
| GAPDHfw1 | AAAGTAAAGGAGGCCGCTGT |
| GAPDHrv1 | CAGCAGCATACGAAGATGGA |
| RPS18fw1 | ACGGTGAAAATCCAGTTTGG |
| RPS18rv1 | GGACACGGATTCCCAGTAGA |

**S2 Table.** **Primer sequences for qPCR of protease contigs.**
