# Supplementary material for: Sugarcane Giant Borer Transcriptome Analysis and Identification of Genes Related to Digestion
Source: PLoS One. 2015 Feb 23;10(2):e0118231. doi: 10.1371/journal.pone.0118231 (PMC4338194; doi:10.1371/journal.pone.0118231)
Supplement: S3 Table — (DOCX) [file pone.0118231.s007.docx]

**S3 Table.** **Top protein domains found in *Telchin licus licus* transcriptome after BLASTx against *Manduca sexta* midgut proteins.**

| **InterPro** | **Frequency** | **Description** |
| --- | --- | --- |
| IPR016040 | 139 | NAD(P)-binding domain |
| IPR001254 | 116 | Peptidase S1/S6, chymotrypsin/Hap |
| IPR001314 | 100 | Peptidase S1A, chymotrypsin |
| IPR009003 | 96 | Serine/cysteine peptidase, trypsin-like |
| IPR016196 | 88 | Major facilitator superfamily, general substrate transporter |
| IPR000618 | 77 | Insect cuticle protein |
| IPR002198 | 72 | Short-chain dehydrogenase/reductase SDR |
| IPR011009 | 71 | Protein kinase-like domain |
| IPR002347 | 67 | Glucose/ribitol dehydrogenase |
| IPR012336 | 66 | Thioredoxin-like fold |
| IPR001128 | 59 | Cytochrome P450 |
| IPR015943 | 59 | WD40/YVTN repeat-like-containing domain |
| IPR011046 | 57 | WD40 repeat-like-containing domain |
| IPR012335 | 55 | Thioredoxin fold |
| IPR013781 | 54 | Glycoside hydrolase, subgroup, catalytic core |
| IPR017853 | 54 | Glycoside hydrolase, catalytic core |
| IPR000504 | 53 | RNA recognition motif, RNP-1 |
| IPR012677 | 53 | Nucleotide-binding, alpha-beta plait |
| IPR011701 | 48 | Major facilitator superfamily MFS-1 |
| IPR017442 | 48 | Serine/threonine-protein kinase-like domain |
| IPR017973 | 47 | Cytochrome P450, C-terminal |
| IPR019781 | 47 | WD40 repeat, subgroup |
| IPR002018 | 46 | Carboxylesterase, type B |
| IPR000834 | 43 | Peptidase M14, carboxypeptidase A |
