# Supplementary material for: Sugarcane Giant Borer Transcriptome Analysis and Identification of Genes Related to Digestion
Source: PLoS One. 2015 Feb 23;10(2):e0118231. doi: 10.1371/journal.pone.0118231 (PMC4338194; doi:10.1371/journal.pone.0118231)
Supplement: S4 Table — (DOCX) [file pone.0118231.s008.docx]

**S4 Table.** **Theoretical physico-chemical parameters of serine proteases identified in SGB transcriptome.**

| **Protein** | **cDNA (bp)** | **ORF (bp)** | **Protein Length (aa)** | **Molecular Mass (kDa)** | **Theoretical pI** | **Putative Protease Type** |
| --- | --- | --- | --- | --- | --- | --- |
| Tl-TRY1 | 880 | 807 | 268 | 28.6 | 5.48 | Trypsin |
| Tl-TRY2 | 1292 | 921 | 306 | 33 | 6.16 | Trypsin |
| Tl-TRY3 | 1043 | 801 | 266 | 28.4 | 6.57 | Trypsin |
| Tl-TRY4 | 898 | 768 | 255 | 27 | 8.79 | Trypsin |
| Tl-TRY5 | 883 | 780 | 259 | 27.8 | 6.5 | Trypsin |
| Tl-CHY1 | 933 | 858 | 285 | 29 | 8.27 | Chymotrypsin |
| Tl-CHY2 | 901 | 840 | 279 | 29.5 | 6.69 | Chymotrypsin |
| Tl-CHY3 | 1033 | 900 | 299 | 32.8 | 6.67 | Chymotrypsin |
| Tl-CHY4 | 1066 | 885 | 294 | 31 | 6.69 | Chymotrypsin |
