# Supplementary material for: Sugarcane Giant Borer Transcriptome Analysis and Identification of Genes Related to Digestion
Source: PLoS One. 2015 Feb 23;10(2):e0118231. doi: 10.1371/journal.pone.0118231 (PMC4338194; doi:10.1371/journal.pone.0118231)
Supplement: S5 Table — (DOCX) [file pone.0118231.s009.docx]

**S5 Table.** **Amino acid sequence identity among SGB APNs.**

| **Protein** | **TlAPN1** | **TlAPN3** | **TlAPN4** |
| --- | --- | --- | --- |
| TlAPN1 | - | 44% | 37% |
| TlAPN3 | - | - | 34% |
| TlAPN4 | - | - | - |
