# Supplementary material for: Sugarcane Giant Borer Transcriptome Analysis and Identification of Genes Related to Digestion
Source: PLoS One. 2015 Feb 23;10(2):e0118231. doi: 10.1371/journal.pone.0118231 (PMC4338194; doi:10.1371/journal.pone.0118231)
Supplement: S6 Table — (DOCX) [file pone.0118231.s010.docx]

**S6 Table.** **Glycosylation sites predicted for SGB APNS.**

| **Protein** | **N-glycosylation (residues)** | **Total** | **O-glycosylation (residues)** | **Total** |
| --- | --- | --- | --- | --- |
| TlAPN1 | 123, 226, 626, 718 | 4 | 48, 49, 57, 704 | 4 |
| TlAPN3 | 121, 175, 228, 270, 673, 746 | 6 | 25, 29, 42, 43, 46, 60, 697, 881, 949, 950, 951, 952, 953, 955, 959, 960, 961, 963 | 18 |
| TlAPN4 | 82, 217, 376, 422, 768, 780 | 6 | 417, 491 | 2 |
